# Multifunctional Fe‐Doped MOF‐808 Nanocomposites for Chemo/Chemodynamic Synergistic Therapy

**DOI:** 10.1002/smll.202512728

**Published:** 2025-11-28

**Authors:** Yang Wang, Ying Pan, Stephen Sproules, Jianqiang Liu, Ross S. Forgan

**Affiliations:** ^1^ School of Pharmaceutical Sciences Zhengzhou University Zhengzhou 450001 China; ^2^ School of Chemistry University of Glasgow Glasgow G12 8QQ UK; ^3^ School of Pharmacy Guangdong Medical University Dongguan Guangdong 523808 China

**Keywords:** chemodynamic therapy, drug delivery, magnetic resonance imaging, metal doping, MOF‐808, synergistic therapy

## Abstract

Theranostic nanomedicines combining targeted drug delivery and emerging therapeutic modalities with diagnosis are a rapidly evolving field in cancer therapy. In addition to conventional drug delivery, metal–organic frameworks (MOFs), consisting of metal ions/clusters and multitopic organic ligands, have been recently highlighted as ideal candidates for chemodynamic therapy (CDT) where active metal ions can be integrated at inorganic secondary building units or coordinated to linkers to act as CDT catalysts. Metal doping provides a potential approach to endow archetypal MOFs with enhanced CDT efficacy; herein, a novel PEGylated Fe‐doped MOF‐808 is reported, which is embedded with ultrasmall gold nanoparticles and manganese ions, can deliver carboplatin (CA) and achieve chemotherapeutic‐chemodynamic (CT/CDT) synergistic therapy. 2D and 3D in vitro experiments demonstrate the synergistic therapeutic efficacy of CT and CDT, while the nanocomposite also shows magnetic resonance imaging potential. Therefore, this work provides a novel protocol to construct multi‐metal doped MOFs as promising candidates in diagnostic and multimodal therapeutic applications.

## Graphical abstract

Fe‐doped MOF‐808 is prepared by a simple, one‐pot synthesis, and sequentially functionalized with Mn ions, ultrasmall gold nanoparticles, and poly(ethylene glycol). Carboplatin‐loaded nanocomposites are capable of synergistic chemotherapy/chemodynamic therapy in 2D and 3D cell cultures, with each added functionality enhancing these effects through specific mechanisms, while the paramagnetic metal ions facilitate visibility by MRI.

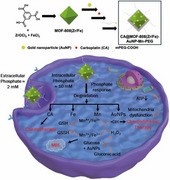

## 1 Introduction

Cancer, a collection of diseases with high morbidity and mortality, has long been categorized as a serious problem which threatens human health.^[^ ^1^ ^]^ As part of a multi‐pronged approach to develop new potential treatments beyond conventional chemotherapy, numerous types of novel therapeutic modes have been reported and studied in depth, including photothermal,^[^ ^2^, ^3^ ^]^ photodynamic,^[^ ^3^, ^4^ ^]^ gas,^[^ ^5^, ^6^ ^]^ and sonodynamic therapies,^[^ ^7^, ^8^ ^]^ wherein some external stimulation is an essential requirement to achieve cytotoxicity.

In cancer, the tumor microenvironment (TME) differs significantly from that of healthy cells, and exhibits unique physical properties, including acidity, the overproduction of hydrogen peroxide (H_2_O_2_), and high concentrations of glutathione (GSH) and phosphate.^[^ ^9^, ^10^ ^]^ Based on these characteristics, the TME provides a suitable environment for non‐invasive chemodynamic therapy (CDT), which utilizes transition metals (e.g., Fe, Co, Ni, Cu, and Mn) as catalysts to disproportionate endogenous H_2_O_2_ and so trigger high‐toxicity hydroxyl radical (•OH) generation via Fenton or Fenton‐like chemistry.^[^ ^11^, ^12^, ^13^ ^]^ Ultrasmall gold nanoparticles can further enhance the conditions for effective CDT, by catalysing the oxidation of glucose to H_2_O_2_ and gluconic acid across a range of conditions, thereby lowering the pH (Fe‐catalysed Fenton chemistry has an optimal reaction pH of 3–4) and increasing H_2_O_2_ concentration.^[^ ^14^ ^]^ Due to its attractive properties, CDT has become a promising anticancer therapeutic strategy, with rapid development in nanomedicine, often alongside alternative treatment modalities.^[^ ^15^ ^]^

Metal–organic frameworks (MOFs), a class of crystalline hybrid materials composed of metal ions/clusters and multitopic organic ligands,^[^ ^16^ ^]^ have been extensively designed as versatile nanoplatforms for drug delivery and nanomedicine in general owing to their multifunctionality, tailorable structure and composition, and tunable pore size.^[^ ^17^, ^18^, ^19^, ^20^, ^21^, ^22^, ^23^, ^24^, ^25^, ^26^, ^27^, ^28^, ^29^, ^30^, ^31^, ^32^ ^]^ Considering metal ions are a core structural feature, MOFs offer the possibility of building a carrier with intrinsic CDT functionality,^[^ ^33^, ^34^, ^35^ ^]^ but those linked by the metals that promote Fenton or Fenton‐like chemistry often exhibit poor chemical stability, and the metal ions themselves have their own toxicity profiles. In contrast, MOFs linked by tetravalent Zr^4+^ offer both chemical stability, stemming from the robustness of the Zr_6_ and Zr_12_ secondary building units (SBUs) comprised of strong metal‐oxide bonds,^[^ ^36^, ^37^, ^38^, ^39^ ^]^ and high biocompatibility.^[^ ^17^ ^]^ We have shown that MOF‐808, a typical zirconium‐based MOF with ideal formula [Zr_6_O_4_(OH)_4_(BTC)_2_(HCOO)_6_] where BTC = benzene‐1,3,5‐tricarboxylate,^[^ ^40^ ^]^ can target specific cancer cell lines through careful modification of its surface chemistry enabled by the labile monocarboxylic acid linkers at its SBUs, while delivering cargo through controlled degradation induced by endogenous and exogenous phosphate, which exhibits a very high affinity for the Zr clusters.^[^ ^17^, ^41^ ^]^ These factors, as well as its larger pore volume and consequent drug loading capacity, lay the foundation for drug delivery applications utilising MOF‐808, even though it has been the subject of considerably less study^[^ ^42^, ^43^, ^44^, ^45^ ^]^ than the archetypal Zr MOF, UiO‐66.^[^ ^46^ ^]^

Metal doping, the deliberate incorporation of extrinsic metal ions into defined SBUs during synthesis,^[^ ^47^, ^48^, ^49^, ^50^, ^51^, ^52^, ^53^ ^]^ offers a route to controllably incorporate small amounts of chemodynamic metal ions into intrinsically stable extant MOF structures. Metal‐doped MOFs typically maintain the structural properties of the pristine MOFs, like their porous structure and large surface area, but often exhibit new functionalities or magnify existing properties,^[^ ^54^, ^55^ ^]^ while offering fine synthetic control over the levels of active metal dopant. In recent studies, metal‐doped MOFs were designed and constructed as versatile theranostic vectors for CDT,^[^ ^56^, ^57^ ^]^ photothermal,^[^ ^58^ ^]^ photodynamic,^[^ ^59^ ^]^ and photoacoustic therapies,^[^ ^60^ ^]^ as well as magnetic resonance imaging (MRI).^[^ ^61^ ^]^

**Scheme 1:**
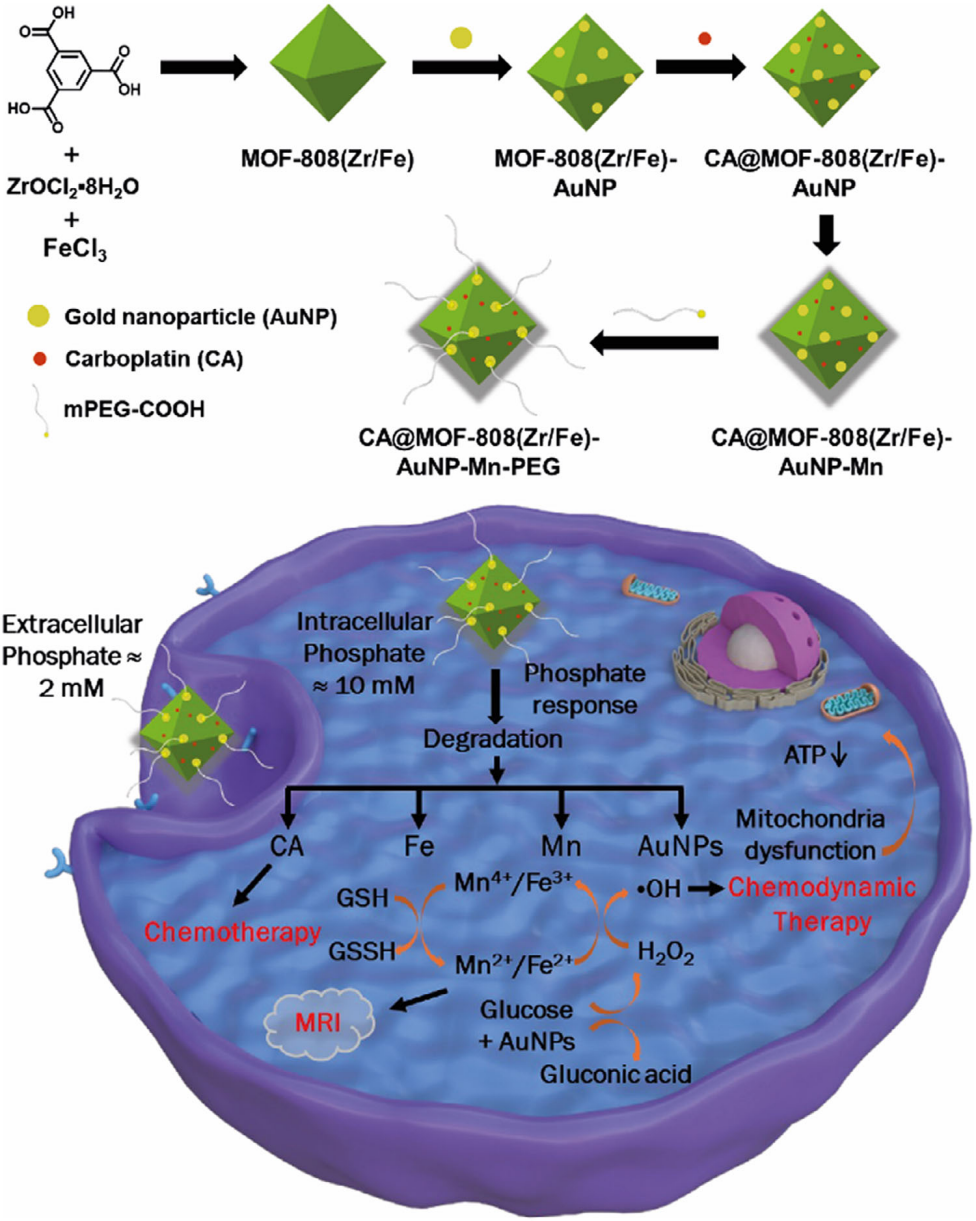
Fabrication of CA@MOF‐808(Zr/Fe)‐AuNP‐Mn‐PEG and schematic illustration of CA@MOF‐808(Fe)‐AuNP‐Mn‐PEG for phosphate‐responsive drug release and chemo/chemodynamic synergistic therapy.

Herein, we describe the synthesis of iron‐doped MOF‐808, designated MOF‐808(Zr/Fe), as a theranostic nanocomposite to achieve synergistic chemo‐ / chemodynamic therapy (CT/CDT) and magnetic resonance imaging. MOF‐808 is highly amenable to modification^[^ ^62^ ^]^ and has previously been doped with Fe for adsorption applications.^[^ ^63^, ^64^ ^]^ Following the incorporation of ultrasmall gold nanoparticles (AuNPs) onto MOF‐808(Zr/Fe), we proceeded to load the chemotherapeutic drug carboplatin (CA) into the nanocarrier. Thereafter, manganese ions were coordinated to its unsaturated metal sites, and its surface was functionalized with carboxylic acid‐terminated polyethylene glycol (mPEG‐COOH), resulting in the formation of the multifunctional nanocomposite CA@MOF‐808(Zr/Fe)‐AuNP‐Mn‐PEG (**Scheme** **1**). After accumulation in cancer cells, dissociation of Mn and Fe ions is expected to catalyse formation of •OH from H_2_O_2_ to achieve CDT, while AuNPs are included to promote the catalytic oxidation of glucose to produce H_2_O_2_ and gluconic acid, enhancing the chemodynamic effect; these are in addition to the release of CA for conventional chemotherapy. Meanwhile, the paramagnetic Mn ions act as MRI contrast reagents to realize the integration of diagnosis and treatment. The nanocomposites exhibit synergistic anti‐tumor efficacy and highlight the potential of metal‐doped MOFs in nanomedicine.

## 2 Results and Discussion

### 2.1 Synthesis and Characterisation of CA@MOF‐808(Zr/Fe)‐AuNP‐Mn‐PEG

**Figure 1:**
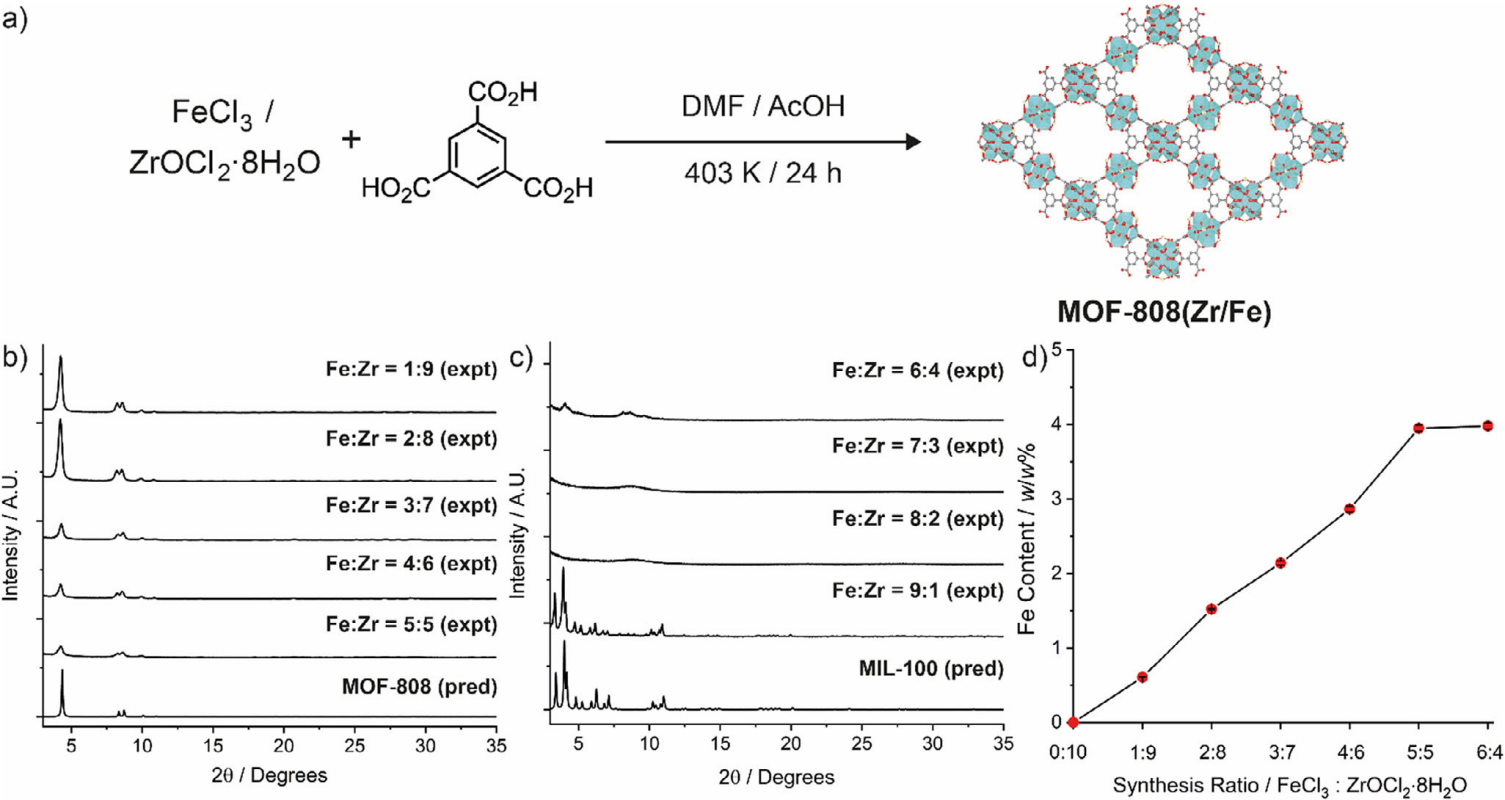
a) Synthetic scheme for Fe‐doped MOF‐808. b) and c) Stacked powder X‐ray diffractograms of samples prepared with differing Fe:Zr ratios in comparison to predicted diffractograms for MOF‐808 and MIL‐100. d) Fe incorporation (% *w*/*w*) in the solids obtained that exhibited Bragg reflections associated with the MOF‐808 structure.

Fe‐doped MOF‐808 was synthesized (Section S2, Supporting Information) by a solvothermal procedure in *N*,*N*‐dimethylformamide, where FeCl_3_ and ZrOCl_2_·8H_2_O were combined with benzene‐1,3,5‐tricarboxylic acid as ligand and acetic acid as modulator (**Figure** **1**a). The iron content in MOF‐808(Zr/Fe) can be controlled by adjusting the molar ratios of FeCl_3_ and ZrOCl_2_·8H_2_O during the synthesis. As the proportion of FeCl_3_ added during the synthesis process was increased (from 10% to 60% of the total stoichiometric metal content), the intensities of Bragg reflections in powder X‐ray diffractograms were gradually reduced, but the crystallization of MOF‐808 was still evident (Figure 1b). Increasing the proportion of FeCl_3_ to 70% and 80% resulted in the disappearance of any Bragg peaks – the isolated solids were amorphous – and when the proportion of FeCl_3_ reached 90%, the diffractogram indicated the formation of the Fe‐BTC MOF, MIL‐100(Fe) (Figure 1c). Inductively coupled plasma optical emission spectroscopy (ICP‐OES) analysis showed a steady increase in the Fe content (*w/w*) with the reagent stoichiometry, reaching a plateau when the intensity of the Bragg peaks became minimal (Figure 1d; Table S1, Supporting Information). However, the Fe:Zr ratios in the materials indicated preferential incorporation of Zr in the structure, which would be expected given the enhanced Lewis acidity of Zr^4+^ compared to Fe^3+^. The material which had the highest Fe content and still retained the MOF‐808 structure whilst being somewhat semicrystalline (optimal synthetic conditions: FeCl_3_:ZrOCl_2_·8H_2_O = 1:1, Fe content: 3.95%, molar ratio Fe/Zr = 28.1%) was chosen for further study, and referred to here on as MOF‐808(Zr/Fe).

**Scheme 2:**
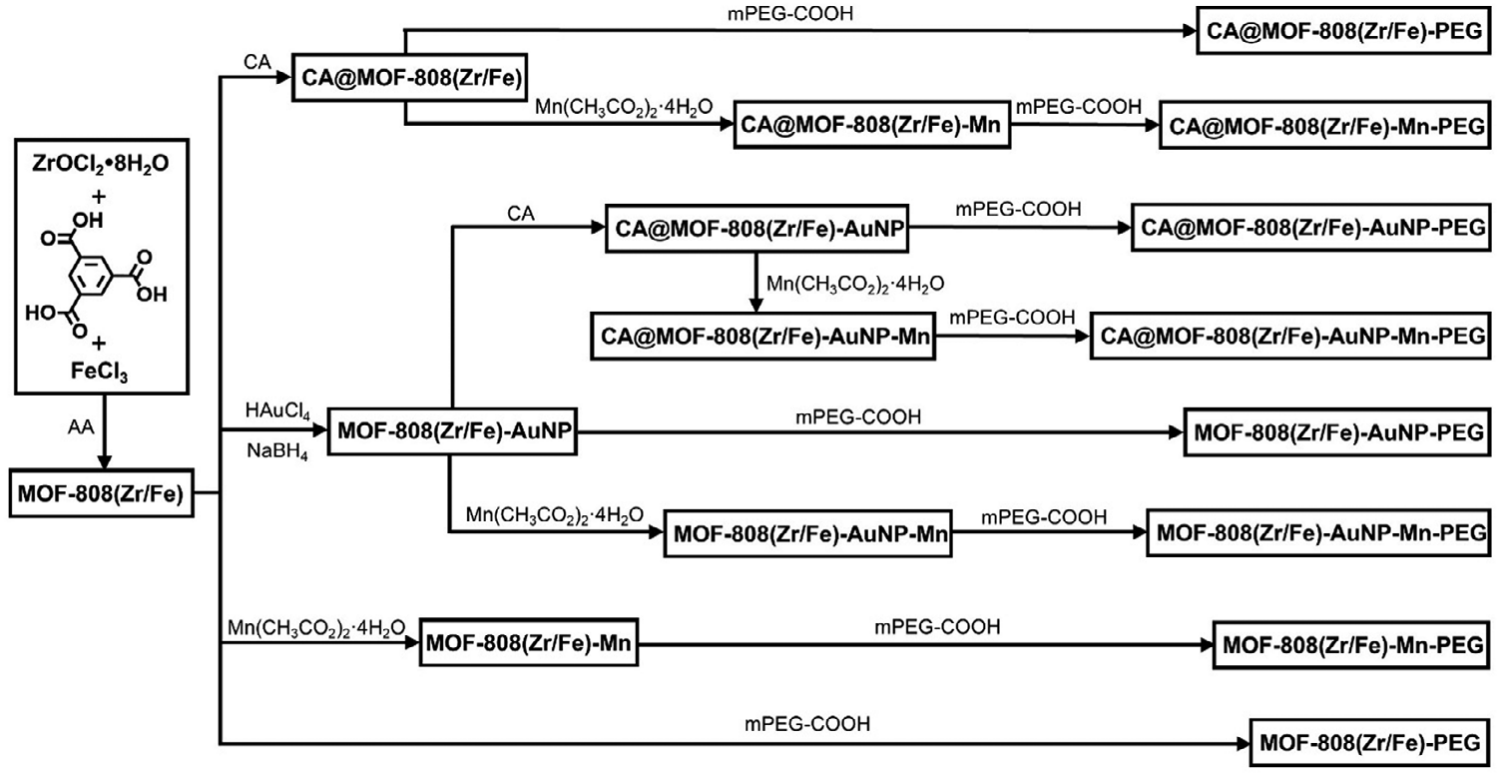
Synthetic scheme for sequential modification of MOF‐808(Zr/Fe).

Following determination of the optimal synthetic conditions for MOF‐808(Zr/Fe), the MOF was sequentially functionalized as outlined in **Scheme** **2**. Ultrasmall gold nanoparticles were grown on the MOF‐808(Zr/Fe) surface by an in situ reduction method,^[^ ^41^ ^]^ using HAuCl_4_ and NaBH_4_, and subsequently, the chemotherapeutic drug carboplatin (CA) was loaded into the pores. Furthermore, manganese sites were immobilized on the MOF by mixing with Mn(CH_3_CO_2_)_2_·4H_2_O via a self‐limiting solvothermal approach,^[^ ^65^ ^]^ and carboxylate‐terminated poly(ethylene glycol)^[^ ^66^ ^]^ was post‐synthetically coated on MOF surface via surface ligand exchange^[^ ^67^ ^]^ to obtain the multifunctional nanocomposite CA@MOF‐808(Zr/Fe)‐AuNP‐Mn‐PEG (the detailed synthetic protocols are given in the Section S2, Supporting Information).

**Figure 2:**
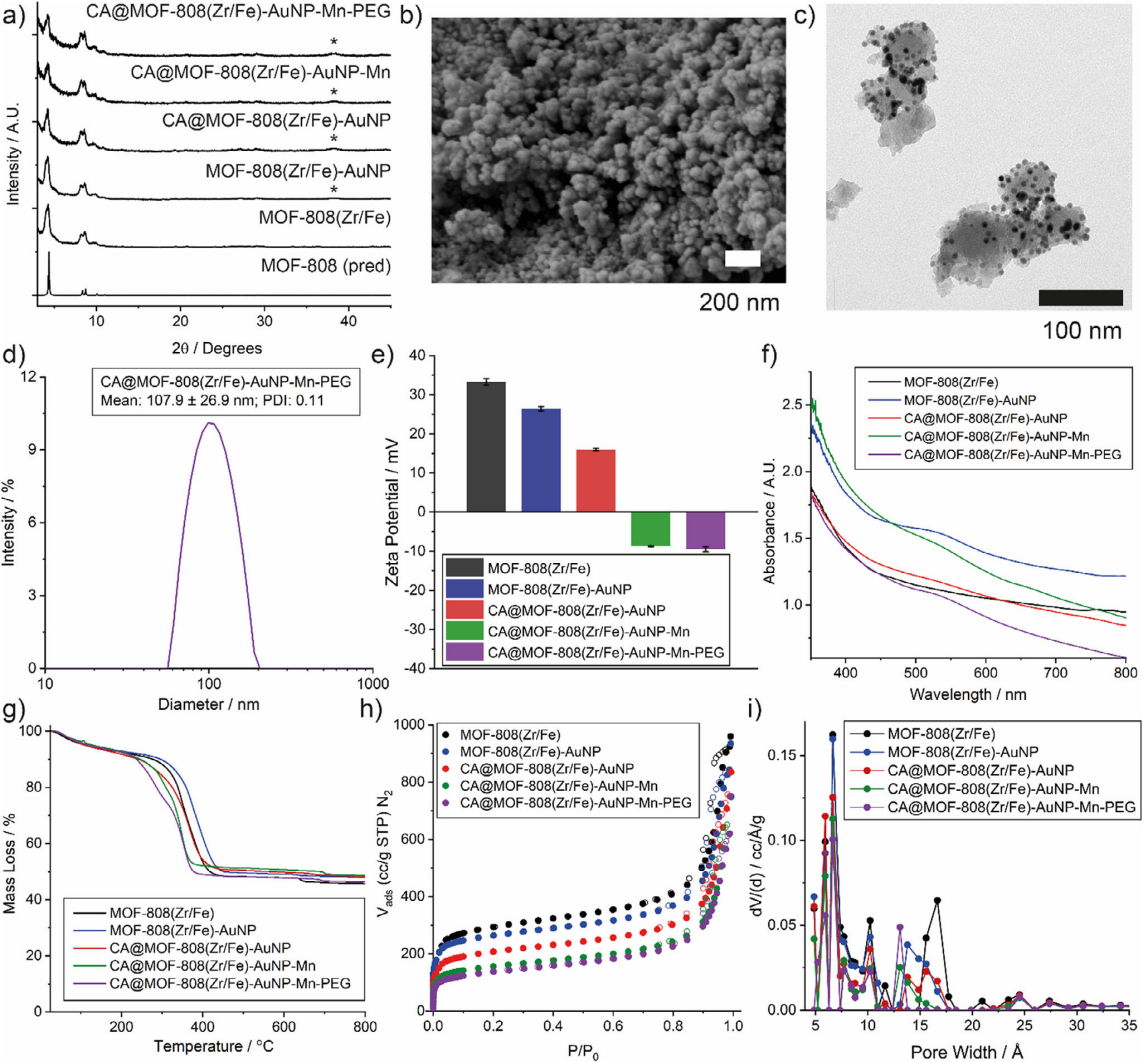
a) Stacked powder X‐ray diffractograms of modified MOF‐808(Zr/Fe) samples compared to the pattern predicted for MOF‐808. Bragg reflections for AuNPs marked with an asterisk. b) SEM and c) TEM images for CA@MOF‐808(Zr/Fe)‐AuNP‐Mn‐PEG; scale bars: 200 and 100 nm, respectively. d) Dynamic light scattering of CA@MOF‐808(Zr/Fe)‐AuNP‐Mn‐PEG in water. e) Comparison of zeta potential measurements for modified MOF‐808(Zr/Fe) in water. f) UV/Vis spectra, g) thermogravimetric analysis traces, h) N_2_ adsorption/desorption isotherms (77 K filled symbols represent adsorption, empty symbols represent desorption), and i) derived pore size distributions (QSDFT, slit cylinder/spherical pore) for the sequentially modified MOF‐808(Zr/Fe) samples.

**Figure** **2** provides indicative characterisation data for the stepwise preparation of CA@MOF‐808(Zr/Fe)‐AuNP‐Mn‐PEG; additional characterisation data for these samples are provided in Section S3.2 (Supporting Information). To ensure the ability to probe the effects of individual components, control samples were also prepared with differing functionalities incorporated, as shown in Scheme 2, with characterisation provided in Section S3.3 (Supporting Information). Stacked PXRD patterns (Figure 2a) demonstrated that CA@MOF‐808(Zr/Fe)‐AuNP‐Mn‐PEG still retains the crystalline structure of MOF‐808 after drug loading and surface modification, and the characteristic Bragg peak of ultrasmall gold nanoparticles ≈2*θ* = 38° was also seen. The morphology and particle size of samples at each stage were characterized by scanning electron microscopy (SEM), showing little difference in size (≈50 nm) or morphology across the samples (a representative image of CA@MOF‐808(Zr/Fe)‐AuNP‐Mn‐PEG is shown in Figure 2b; the remainder are in Figure S3, Supporting Information). Transmission electron microscopy (Figure 2c) showed that ultrasmall gold nanoparticles on MOFs surface of ≈8 nm could be distinctly observed on CA@MOF‐808(Zr/Fe)‐AuNP‐Mn‐PEG, although distribution was uneven across individual MOF nanoparticles. Moreover, dynamic light scattering (DLS) analysis (Figure 2d) showed that the hydrodynamic diameter, polydispersity index (PDI) and zeta potential (Figure 2e) of CA@MOF‐808(Zr/Fe)‐AuNP‐Mn‐PEG are 107.9 ± 26.9 nm, 0.11, and −9.5 mV, respectively, suggesting minimal aggregation in water. The zeta potentials of the samples showed a sequential decrease as functionality was added to the nanocomposite.

A number of methods were used to assess the composition of CA@MOF‐808(Zr/Fe)‐AuNP‐Mn‐PEG. UV–vis spectra (Figure 2f) showed the characteristic peak of ultrasmall gold nanoparticles at ≈520 nm throughout each step. As shown in thermogravimetric analysis traces (Figure 2g), compared with naked MOF‐808(Zr/Fe), drug loading and polymer coating brought the onset of thermal degradation to lower temperatures, while the inorganic residual mass increased after embedding of ultrasmall gold nanoparticles and immobilisation of Mn, as would be expected. N_2_ adsorption isotherms (Figure S2, Supporting Information) showed MeOH activation enhanced the porosity (from 0.36 to 0.44 cc g^−1^) and BET surface area (from 872 to 1078 m^2^ g^−1^) of MOF‐808(Zr/Fe), with sequential decreases in porosity (Figure 2h) and pore volume (Figure 2i) as components were added; CA@MOF‐808(Zr/Fe)‐AuNP‐Mn‐PEG exhibited remarkable drop of porosity (from 0.44 to 0.20 cc g^−1^) and BET area (from 1078 to 498 m^2^ g^−1^) after surface modification and drug loading (Table S2, Supporting Information). These qualitative assessments were confirmed by ICP‐OES, which was employed for quantitative determination of metal doping and CA loading (Table S3, Supporting Information), revealing high Fe doping (3.44 wt%), Mn immobilisation (3.5 wt%), incorporation of AuNPs (1.6 wt%) and CA loading (6.1 wt%).

**Figure 3:**
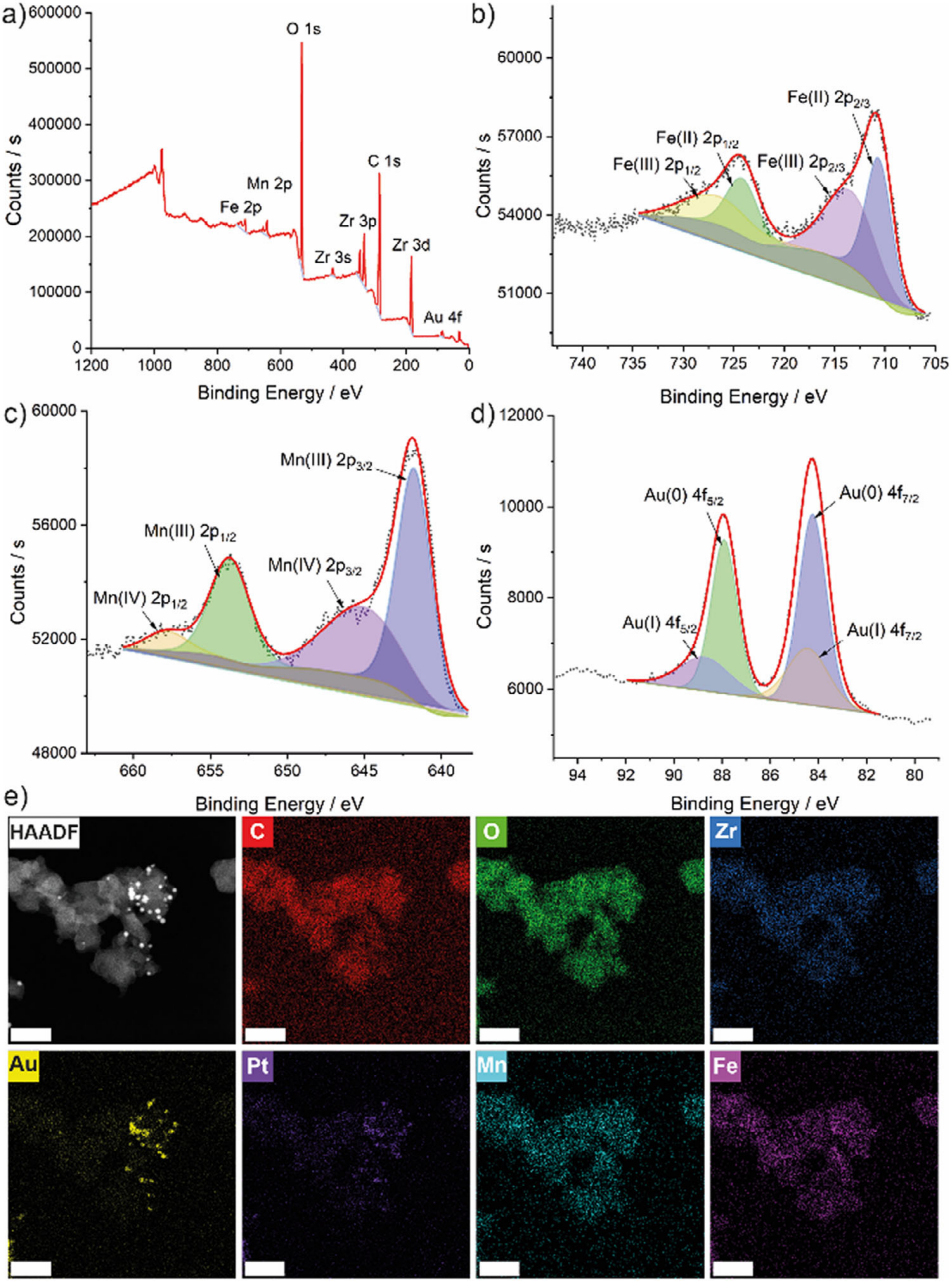
a) The X‐ray photoelectron spectrum of CA@MOF‐808(Zr/Fe)‐AuNP‐Mn‐PEG with portions focussed on b) the Fe 2p region, c) the Au 4f region, and d) the Mn 2p region, with deconvolutions and peak assignments. e) STEM images and corresponding elemental mapping images (C, O, Zr, Au, Pt, Mn and Fe) for CA@MOF‐808(Zr/Fe)‐AuNP‐Mn‐PEG (scale bars: 50 nm).

The X‐ray photoelectron spectroscopy (XPS) spectrum of MOF‐808(Zr/Fe)‐AuNP‐Mn‐PEG was measured (**Figure** **3**a) to reveal the chemical composition and characteristic elements, where the signals of Fe 2p, Mn 2p, C 1s, O 1s, Zr 3d, and Au 4f could be observed (Figure 3b–d; Figure S5, Supporting Information). XPS also allows an assessment of the oxidation states of the potential Fenton catalysts (and MRI contrast agents) at the surface and near surface of CA@MOF‐808(Zr/Fe)‐AuNP‐Mn‐PEG. Specifically, the two characteristic peaks at 710.59 and 724.14 eV were ascribed to Fe^2+^, and 713.44 and 726.61 eV peaks were ascribed to Fe^3+^, where the peak area ratio of Fe^2+^/Fe^3+^ is 0.93 (Figure 3b). Furthermore, the Mn 2p_2/3_ peak could be deconvoluted as Mn^3+^ (641.77 eV) and Mn^4+^ (645.11 eV), where the peak area ratio of Mn^3+^ to Mn^4+^ is 3.90 (Figure 3c). Hence, XPS data suggest that the presence of Fe and Mn provides a foundation for Fenton‐like reaction‐based CDT. The Au 4f spectrum showed strong peaks of Au(0) 4f_7/2_ (84.24 eV) and Au(0) 4f_5/2_ (87.92 eV), demonstrating successful reduction of the Au^3+^ reagents and that the majority of Au exists in the form of AuNP (Figure 3d). Classical elemental mapping during electron microscopy (Figure 3e) complemented the XPS data, confirming the presence of the various elements. Both Fe and Mn were well distributed across the particles, with something of a co‐localisation of Au and Pt, suggesting CA associates in part with the AuNPs.

**Figure 4:**
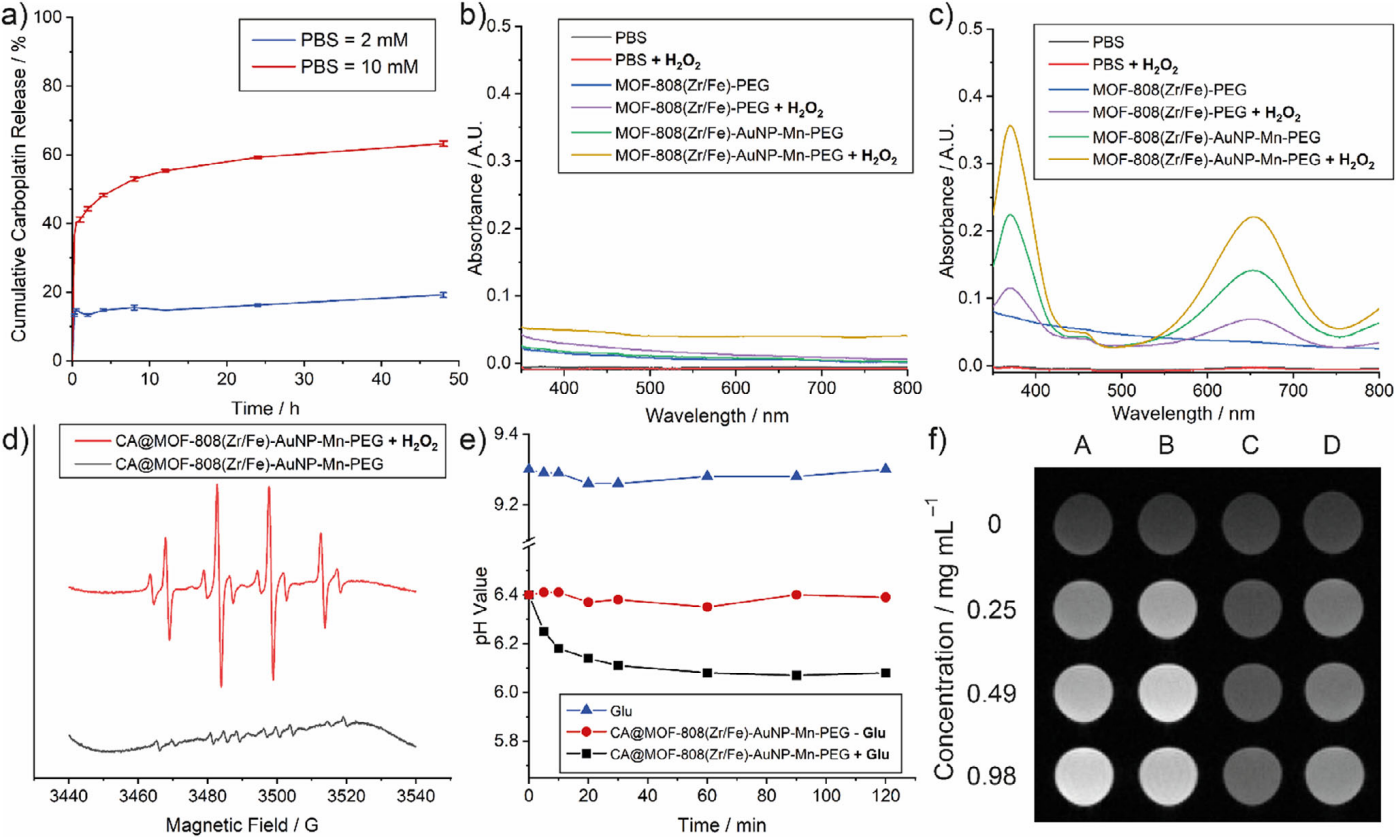
a) Carboplatin (CA) release from CA@MOF‐808(Zr/Fe)‐AuNP‐Mn‐PEG in PBS (pH = 7.4) of different total phosphate concentrations. Data shown are mean of three technical replicates, error bars denote standard deviations. UV–vis spectra of a PBS solution of TMB after different treatments (MOFs concentration: 250 µg mL^−1^, TMB concentration: 50 µg mL^−1^, H_2_O_2_ concentration: 10 mm) at b) pH = 7.4, and c) pH 6.0. d) ESR spectra of PBS solution of CA@MOF‐808(Zr/Fe)‐AuNP‐Mn‐PEG (200 µg mL^−1^) and H_2_O_2_ (10 mM) + CA@MOF‐808(Zr/Fe)‐AuNP‐Mn‐PEG (200 µg mL^−1^) at pH 6.0, using DMPO as the spin trap. e) The pH values of aqueous suspensions of CA@MOF‐808(Zr/Fe)‐AuNP‐Mn‐PEG reacted with glucose at various times (MOF concentration: 250 µg mL^−1^, glucose concentration: 2 mg mL^−1^). f) *T*_1_‐weighted MRI images of different concentrations CA@MOF‐808(Zr/Fe)‐AuNP‐Mn‐PEG under different conditions (A: pH 5.0, GSH: 0 mm; B: pH 5.0, GSH: 10 mm; C: pH 7.4, GSH: 0 mm, D: pH 7.4, GSH: 10 mm).

To characterize drug delivery potential, the release of CA from CA@MOF‐808(Zr/Fe)‐AuNP‐Mn‐PEG into different concentrations of phosphate‐buffered saline (PBS) was analysed by ICP‐OES. As shown in **Figure** **4**a, only 19.2% of CA was released in PBS (2 mm) within 48 h. On the contrary, 63.2% of CA was burst released in the 10 mm PBS within 48 h, owing to the high binding affinity of phosphate to Zr cluster compared with carboxylate ligands, leading to the degradation of the nanocomposite at higher phosphate concentrations, which mimic intracellular conditions^[^ ^41^ ^]^ and provide a trigger for intracellular CA release. To assess the potential for CDT, the generation of hydroxyl radicals (•OH) was investigated via 3,3′,5,5′‐tetramethylbenzidine (TMB) assay in PBS, where TMB can capture •OH and be oxidized (to oxidized TMB, oxTMB), which exhibits maximum absorbance at λ = 652 nm. There was no notable absorbance ascribed to oxTMB across the samples assessed in PBS at pH 7.4 (Figure 4b), other than some absorbance induced by MOF‐808(Zr/Fe)‐AuNP‐Mn‐PEG, which we attribute to the mixed valence Mn^3+^/Mn^4+^ inducing residual oxidation of TMB. In contrast, MOF‐808(Zr/Fe)‐PEG showed some absorbance from oxTMB in a tumor‐mimic environment (10 mm H_2_O_2_, pH 6.0), indicating the generation of •OH (Figure 4c), while MOF‐808(Zr/Fe)‐AuNP‐Mn‐PEG showed significantly enhanced absorbance of oxTMB, which is attributed to the co‐catalytic ability of Fe and Mn for Fenton and Fenton‐like reactions. To determine the type of generated radical, electron spin resonance (ESR) spectroscopy was conducted, which showed the typical 1:2:2:1 signal indicating •OH production (Figure 4d) by MOF‐808(Zr/Fe)‐AuNP‐Mn‐PEG that was trapped by 5,5‐dimethyl‐1‐pyrroline‐*N*‐oxide (DMPO). The glucose oxidase‐mimicking activity of the ultrasmall gold nanoparticles was also studied. Glucose can be oxidized by ultrasmall gold nanoparticles to produce H_2_O_2_ and gluconic acid, decreasing the solution pH value. As shown in Figure 4e, the pH value of an aqueous suspension of CA@MOF‐808(Zr/Fe)‐AuNP‐Mn‐PEG decreased from 6.40 to 6.08 after incubation with glucose, while there was no pH change in CA@MOF‐808(Zr/Fe)‐AuNP‐Mn‐PEG or glucose alone. CA@MOF‐808(Zr/Fe)‐AuNP‐Mn‐PEG is expected to degrade in response to the high intracellular phosphate concentrations found in tumor‐mimic microenvironments, thereby releasing Mn ions, typical *T*_1_ contrast agents, for *T*_1_‐weighted magnetic resonance imaging. As shown in Figure 4f, under the simulated physiological conditions (pH 7.4, column C in Figure 4f), the nanocomposite exhibited very poor MRI performance, while under simulated tumor microenvironment (pH 5.0, GSH = 10 mm, column B in Figure 3f), the MRI showed a remarkable whitening effect that reached a maximum at a concentration of 0.49 mg mL^−1^. These results demonstrate that CA@MOF‐808(Zr/Fe)‐AuNP‐Mn‐PEG also has potential as an MRI contrast agent, and therefore as a theranostic composite.

### 2.2 Cellular Uptake, Intracellular •OH Generation, and In Vitro Cytotoxicity

**Figure 5:**
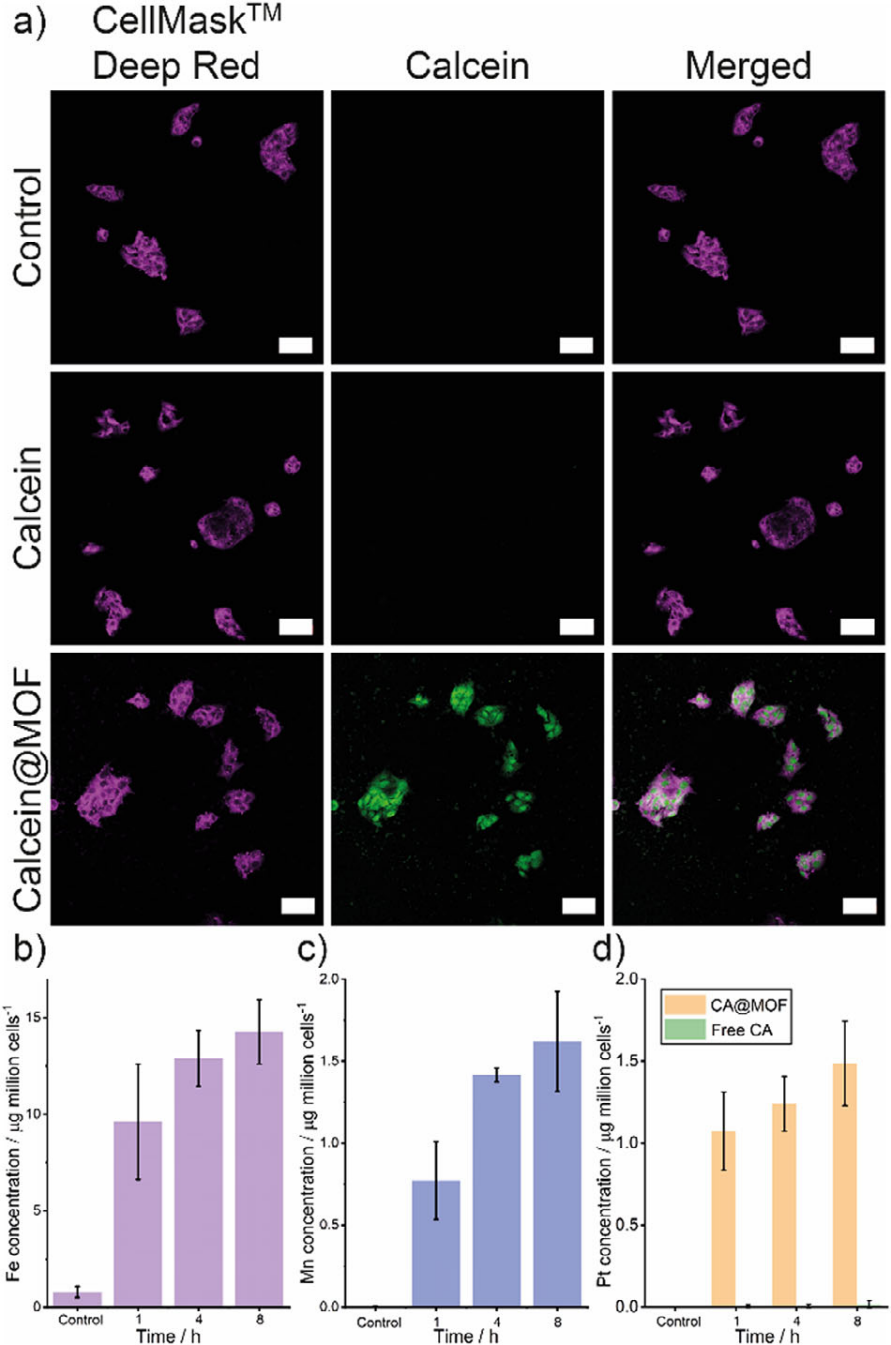
a) CLSM images of HepG2 cells incubated with Cal@MOF‐808(Zr/Fe)‐AuNP‐Mn‐PEG for 6 h (concentration: 50 µg mL^−1^; CellMask^TM^ Deep Red: λ_ex_ = 633 nm, λ_em_ = 669 nm; calcein: λ_ex_ = 488 nm, λ_em_ = 530 nm; scale bars: 50 µm). Intracellular b) Mn and c) Fe content of HepG2 cells treated with CA@MOF‐808(Zr/Fe)‐AuNP‐Mn‐PEG at different times. d) Intracellular Pt content of HepG2 cells treated with CA@MOF‐808(Zr/Fe)‐AuNP‐Mn‐PEG and free CA at different times (MOF concentration: 50 µg mL^−1^, CA concentration: 3 µg mL^−1^). Data shown are mean values of three biological replicates, error bars denote standard deviations.

Initial experiments assessed the chemodynamic and chemotherapeutic effects of the nanocomposites in vitro (Section S4, Supporting Information). The cellular uptake of this nanocomposite was evaluated with confocal laser scanning microscopy (CLSM) using Cal@MOF‐808(Zr/Fe)‐AuNP‐Mn‐PEG, which was prepared using calcein (Cal) to label the nanocomposite with 11.5% loading capacity (Figure S12, Supporting Information). As shown in **Figure** **5**a, after 6 h incubation with Cal@MOF‐808(Zr/Fe)‐AuNP‐Mn‐PEG, the strong fluorescence intensity of Cal was observed in human hepatocellular carcinoma (HepG2) cells, which demonstrates the nanocomposite was internalized by HepG2 cells. The negligible cellular uptake of free Cal is ascribed to its hydrophilicity, which we have observed previously.^[^ ^68^, ^69^ ^]^

Furthermore, ICP‐OES was also conducted to investigate the cellular uptake behavior by incubating HepG2 cells with CA@MOF‐808(Zr/Fe)‐AuNP‐Mn‐PEG. As shown in Figure 5b,c, with the extension of incubation time, the content of Fe and Mn gradually increased in HepG2 cells, which demonstrates that HepG2 cells could effectively uptake the nanocomposite. The intracellular Pt concentration (stemming from CA) was determined to evaluate the CA delivery efficacy (Figure 5d). It was worth noting that CA@MOF‐808(Zr/Fe)‐AuNP‐Mn‐PEG showed a ≈100‐fold increase in Pt content compared to free CA, which confirms MOF‐808(Zr/Fe)‐AuNP‐Mn‐PEG is a satisfactory nanocarrier for CA delivery to improve the accumulation of CA in cancerous cells.

**Figure 6:**
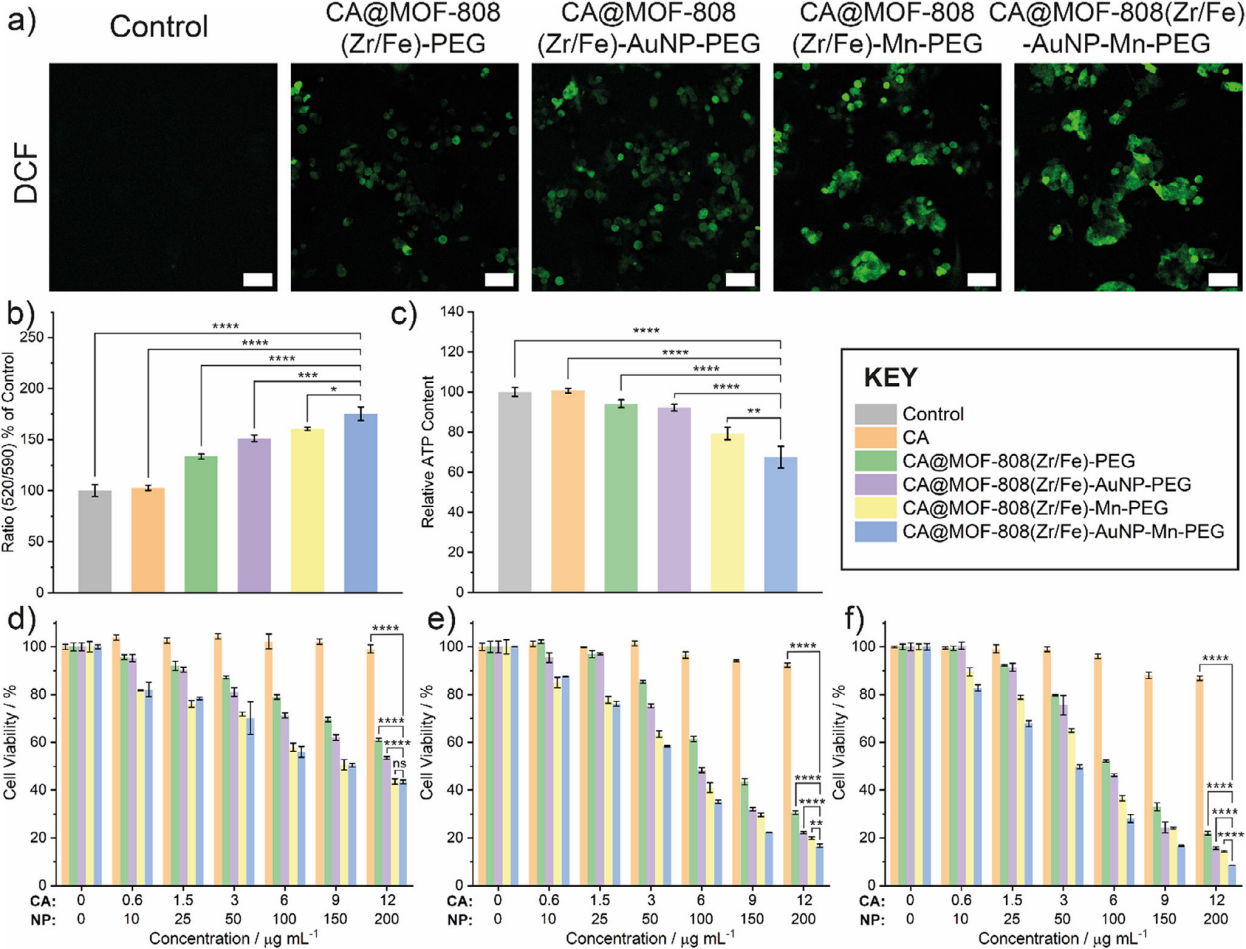
a) CLSM images of oxidized DCF fluorescence in HepG2 cells with different MOF‐808(Zr/Fe) samples for 24 h incubation (concentration: 200 µg mL^−1^; DCF: λ_ex_ = 488 nm, λ_em_ = 535 nm; scale bars: 50 µm). b) Mitochondrial membrane potential changes in HepG2 cells with different MOF‐808(Zr/Fe) samples for 24 h incubation (concentration: 200 µg mL^−1^; JC‐aggr: λ_ex_ = 540 nm, λ_em_ = 590 nm, cut off at 570 nm; JC‐mono: λ_ex_ = 490 nm, λ_em_ = 525 nm, cut off at 515 nm; n = 3). c) Relative ATP content in HepG2 cells with different MOF‐808(Zr/Fe) samples for 24 h incubation (concentration: 200 µg mL^−1^, n = 3). Cell viabilities of HepG2 cells incubated with different CA‐loaded MOF‐808(Zr/Fe) samples for d) 24 h, e) 48 h, and f) 72 h. The concentration of CA corresponds to that of the free drug or that delivered by the MOF (assumes a standard CA loading of 6%, actual loadings are 4.9% for CA@MOF‐808(Zr/Fe)‐PEG, 7.6% for CA@MOF‐808(Zr/Fe)‐AuNP‐PEG, 5.0% for CA@MOF‐808(Zr/Fe)‐Mn‐PEG and 6.2% for CA@MOF‐808(Zr/Fe)‐AuNP‐Mn‐PEG; line graphs against CA concentration are given in the Figure S14, Supporting Information). Data shown are mean of three biological replicates, error bars denote standard deviations. Statistical significance of 200 µg mL^−1^ dose calculated by one‐way analysis of variance (ANOVA): *p <0.05, **p <0.01, ***p <0.001, ****p <0.0001, and ns means not significant.

To investigate the intracellular •OH generation of the nanocomposite, CLSM was conducted using 2′,7′‐dichlorofluorescein diacetate (DCFH‐DA) as a specific indicator, which can be hydrolysed by intracellular esterase and oxidized by •OH to convert to dichlorofluorescein (DCF) with green fluorescence. As shown in **Figure** **6**a, CA@MOF‐808(Zr/Fe)‐PEG induced green fluorescence in HepG2 cells incubated with DCFH‐DA, which demonstrates that Fe‐doped MOF‐808 can act as a catalyst for Fenton reaction‐based CDT. After AuNP incorporation, CA@MOF‐808(Zr/Fe)‐AuNP‐PEG showed a slight improvement in the fluorescent intensity of DCF owing to extra H_2_O_2_ generation through the oxidation of intracellular glucose by glucose oxidase‐mimicking AuNP. Notably, CA@MOF‐808(Zr/Fe)‐AuNP‐Mn‐PEG, with manganese ions immobilized, exhibited stronger intracellular green fluorescence, which corresponds to a greater extent of •OH generation resulting from the combination of Fe and Mn delivering dual catalytic ability to improve CDT efficacy. Mitochondrial membrane potential changes in HepG2 cells were assessed by the JC‐10 assay, which is typically used as a marker of mitochondrial damage. As shown in Figure 6b, after CA@MOF‐808(Zr/Fe)‐AuNP‐Mn‐PEG treatment, the ratio of JC‐mono and JC‐aggr notably increased compared with the control group, indicating the nanocomposite causes depolarization of the membrane potential across the mitochondria. Meanwhile, the relative intracellular adenosine triphosphate (ATP) content was evaluated by luminescent ATP detection assay, which showed decreased ATP levels following the above treatment, further confirming the mitochondrial dysfunction (Figure 6c).

The cytotoxicity of free CA, CA‐loaded and CA‐free MOF‐808(Zr/Fe) nanocomposites against 2D HepG2 cell cultures was evaluated by the AlamarBlue assay at different time periods (Figure 6d–f). As expected, free CA exhibited negligible cytotoxicity, ascribed to low intracellular uptake,^[^ ^44^, ^45^ ^]^ which was confirmed by the intracellular ICP‐OES data described above. In contrast, all the MOF‐808(Zr/Fe) nanocomposites raised cytotoxicity in a dose‐dependent manner owing to the enhanced bioavailability of CA. According to calculated IC_50_ (half maximal inhibitory concentrations) data (Table S5, Supporting Information), CA@MOF‐808(Zr/Fe)‐AuNP‐Mn‐PEG (IC_50_ = 44.7 ± 1.0 µg mL^−1^) showed obvious enhancement in cancer killing efficacy compared with other control groups (103.4 ± 1.0 µg mL^−1^ for CA@MOF‐808(Zr/Fe)‐PEG; 89.0 ± 1.0 µg mL^−1^ for CA@MOF‐808(Zr/Fe)‐AuNP‐PEG; 68.7 ± 1.0 µg mL^−1^ for CA@MOF‐808(Zr/Fe)‐Mn‐PEG), which may indicate the doping of Mn and Fe can achieve the co‐catalysis of Fenton/Fenton‐like reactions, and incorporation of AuNP can catalyse the oxidation of glucose to H_2_O_2_ and gluconic acid. Furthermore, the CDT efficacy of the empty nanocomposites themselves was also evaluated, and the IC_50_ values were calculated (Figure S13 and Table S5, Supporting Information), where MOF‐808(Zr/Fe)‐AuNP‐Mn‐PEG showed the best cancer killing efficacy with IC_50_ = 147.4 ± 1.0 µg mL^−1^, but at a concentration significantly higher than the CA‐loaded analogue. The IC_50_ values for the other CA‐free MOF‐808(Zr/Fe) nanocomposites were too large to be determined across the experimental concentration range. Then, the biocompatibility of CA@MOF‐808(Zr/Fe)‐AuNP‐Mn‐PEG towards the HEK 293 cells (human embryonic kidney) was also studied, which showed IC_50_ = 27.1 ± 1.01 µg mL^−1^ after 72 h incubation (Figure S15 and Table S5, Supporting Information). Taken together, these in vitro data demonstrate that CA@MOF‐808(Zr/Fe)‐AuNP‐Mn‐PEG can successfully achieve CT/CDT synergistic therapy through delivery of CA and induction of reactive oxygen species.

**Figure 7:**
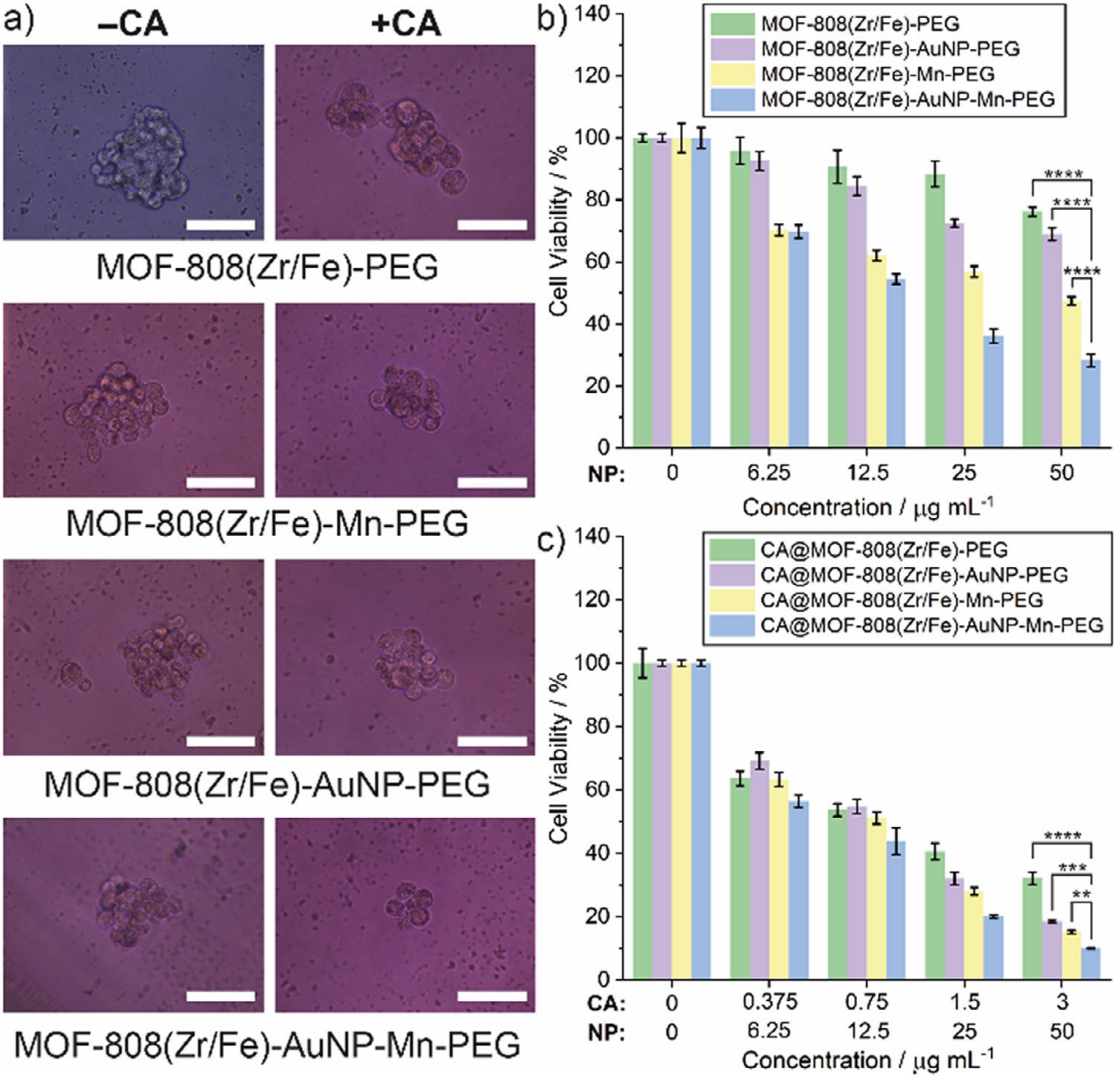
a) Representative light microscopic imaging of HepG2 spheroids incubated with various CA‐free and CA‐loaded MOF‐808(Zr/Fe) samples for 5 days (concentration: 12.5 µg mL^−1^, scale bar: 100 µm). Cell viability of HepG2 spheroids incubated with different concentrations of b) CA‐free and c) CA‐loaded MOF‐808(Zr/Fe) samples for 5 days. The concentration of CA corresponds to that of the free drug or that delivered by the MOF (assumes a standard CA loading of 6%; line graphs against CA concentration are given in Figure S16, Supporting Information). Data shown are mean of three biological replicates, error bars denote standard deviations. Statistical significance of 50 µg mL^−1^ dose calculated by one‐way analysis of variance (ANOVA), **p <0.01, ***p <0.001, ****p <0.0001.

Given that tumorigenic sphere formation can more reliably mimic real tumor than monolayer cell culture,^[^ ^70^, ^71^ ^]^ matrix‐free HepG2 spheroids were prepared to test the in vivo potential of CA@MOF‐808(Zr/Fe)‐AuNP‐Mn‐PEG. As shown in **Figure** **7**a, Among the CA‐free MOF‐808(Zr/Fe) nanocomposites, MOF‐808(Zr/Fe)‐AuNP‐Mn‐PEG most significantly reduced the size of HepG2 spheroids after being incubated with suspended HepG2 cells for 5 days, which indicates that the cumulative effect of AuNP catalysing the oxidation of glucose, and Mn and Fe dual‐catalysing Fenton/Fenton‐like chemistry, promotes the greatest CDT efficacy. Meanwhile, the size of HepG2 spheroids was smaller still after incubation with an identical concentration of CA@MOF‐808(Zr/Fe)‐AuNP‐Mn‐PEG, demonstrating that CT/CDT synergistic therapy improved the anti‐tumor effect. To further investigate the cell viability of HepG2 spheroids, the AlamarBlue assay was used (Figure 7b,c). Among the drug free materials, MOF‐808(Zr/Fe)‐AuNP‐Mn‐PEG showed highest anti‐tumor efficacy towards the spheroids, ascribed to CDT, with an IC_50_ of 15.2 ± 1.1 µg mL^−1^, while the IC_50_ of the other CA‐free MOF‐808(Zr/Fe) nanocomposites was much higher or could not be determined, the same trend as the 2D cell culture results (Table S6, Supporting Information).

Similar trends were observed for the CA‐loaded MOF‐808(Zr/Fe) nanocomposites, where CA@MOF‐808(Zr/Fe)‐AuNP‐Mn‐PEG, possessing the CT/CDT synergistic therapeutic model, exhibited the lowest IC_50_ (8.6 ± 1.1 µg mL^−1^). As with the 2D cultures, free CA showed negligible toxicity to HepG2 spheroids owing to the low uptake by HepG2 cells (Figure S17, Supporting Information). Overall, these results correlate well with the data from 2D in vitro experiments, further highlighting the advantageous dual‐mode properties of CA@MOF‐808(Zr/Fe)‐AuNP‐Mn‐PEG and paving the way for in vivo investigations into biocompatibility and targeting.

## 3 Conclusion

In summary, a novel Fe‐doped MOF‐808‐based nanocomposite, PEGylated MOF‐808(Zr/Fe) with embedded ultrasmall gold nanoparticles and immobilized Mn ions, was constructed and loaded with carboplatin for CT/CDT synergistic therapy. In vitro experiments against 2D and 3D cell cultures confirmed the stepwise increase in chemodynamic efficiency induced by the different functionalisations in the empty carriers. Loading with carboplatin led to a synergistic therapeutic effect against HepG2 cells, in 2D cultures and matrix‐free spheroids, with CA@MOF‐808(Zr/Fe)‐AuNP‐Mn‐PEG being the most potent nanocomposite by a combination of chemotherapy and enhanced CDT efficacy by Mn and Fe co‐catalysed Fenton/Fenton‐like reaction. The chemodynamic effects were confirmed by assessing generation of reactive oxygen species in test tubes and in vitro, inducing mitochondrial dysfunction, and the presence of paramagnetic Mn^2+^ ions allowed preliminary assessment of the nanocomposite's suitability as an MRI contrast agent. This work, therefore, provides a novel strategy for probing the validity of heterometal‐doped MOFs in diagnostic and therapeutic applications, including frontier modalities such as synergistic CT/CDT.

## Conflict of Interest

The authors declare no conflict of interest.

## Supporting information



Supporting Information

## Data Availability

The data that support the findings of this study are available from the corresponding author upon reasonable request.
