# Supplementary material for: Multifunctional Fe‐Doped MOF‐808 Nanocomposites for Chemo/Chemodynamic Synergistic Therapy
Source: Small. 2025 Nov 28;22(4):e12728. doi: 10.1002/smll.202512728 (PMC12809198; doi:10.1002/smll.202512728)
Supplement: Supplementary file 1 — Supporting Information [file SMLL-22-e12728-s001.pdf]

NANO · MICRO
small

Supporting Information

for *Small*, DOI 10.1002/smll.202512728

Multifunctional Fe-Doped MOF-808 Nanocomposites for Chemo/Chemodynamic Synergistic Therapy

Yang Wang, Ying Pan, Stephen Sproules, Jianqiang Liu and Ross S. Forgan**

Multifunctional Fe-doped MOF-808 Nanocomposites for Chemo/Chemodynamic Synergistic Therapy

Yang Wang,[1*] Ying Pan,[1,2] Stephen Sproules,[1] Jianqiang Liu,[2] Ross S. Forgan[1*]

1. School of Chemistry, University of Glasgow, Glasgow G12 8QQ, UK.
Email: yw2669517@163.com; ross.forgan@glasgow.ac.uk
2. School of Pharmacy, Guangdong Medical University, Dongguan 523808, Guangdong, China.

Supporting Information

S1. Materials and Experimental Apparatus. **S2.**
S2. Synthesis, Drug Loading, and Postsynthetic Modification. **S4.**
S3. Characterisation. **S9.**
S4. *In Vitro* Experiments. **S21.**
S5. References. **S30.**

S1. Materials and Experimental Apparatus

Zirconyl chloride octahydrate, iron(III) chloride, 3,3',5,5'-Tetramethylbenzidine, acetic acid, dichloromethane, acetone, and methanol were purchased from Fisher Scientific. Succinic anhydride, 4-(dimethylamino)pyridine, benzene-1,3,5-tricarboxylic acid, manganese(II) acetate tetrahydrate, sodium borohydride, poly(ethylene glycol) methyl ether (MW 2000), 5,5-dimethyl-1-pyrroline *N*-oxide (DMPO) and trichlorogold hydrochloride hydrate were purchased from Alfa Aesar. Hydrochloric acid (36.5%), *N*,*N*-dimethylformamide (DMF) and nitric acid (70%) were purchased from Honeywell. Carboplatin, Dulbecco's Modified Eagle Medium (DMEM), fetal bovine serum (FBS, qualified, Brazil), penicillin-streptomycin (with 10,000 units penicillin and 10 mg streptomycin/mL), L-glutamine (200 mM, 100X), 0.25% trypsin-EDTA (1X), recombinant human EGF lyophilized, B-27 supplement (50X), phosphate buffer saline (PBS, pH 7.4, 1X) and Dulbecco's phosphate-buffered saline (DPBS, pH 7.2, 1X) were purchased from Thermo Fisher Scientific. AlamarBlue™ Cell Viability Reagent, CellMask™ Deep Red Plasma Membrane Stains and ProLong™ Glass Antifade Mountant were obtained from Invitrogen. All reagents were obtained from commercial sources and were used without any further purification.

^{1}H Nuclear Magnetic Resonance Spectroscopy (^{1}H NMR): ^{1}H NMR spectra were collected on a Bruker Avance III 400 MHz spectrometer referenced to residual solvent peaks. All the tests were conducted at 298 K.

Powder X-Ray Diffraction (PXRD): PXRD measurements were tested by a Rigaku MiniFlex benchtop diffractometer equipped with a Cu-sealed tube X-ray source (λ(CuKα) = 1.4505 Å) at 298 K.

Thermogravimetric Analysis (TGA): TGA spectra were carried out using TA Instruments TGA 5500 Thermogravimetric Analyser. Test conditions ranged from room temperature to 1073 K with a 10 K min^{-1} heating rate under an air atmosphere.

Gas Uptake and Pore-Size Distribution: N_2 adsorption isotherms were measured by a Quantachrome Autosorb iQ gas sorption analyser at 77 K. Samples were degassed

under vacuum at 393 K for 20 h by using an internal turbo pump. BET surface areas and pore size distributions were calculated by Micropore BET Assistant in the Quantachrome ASiQwin operating software.

Scanning Electron Microscopy (SEM): The samples were treated with a conductive coating (15 nm layer of 80/20 Au/Pd) by Polaron SC7640 sputter coater and imaged by a Carl Zeiss Sigma Variable Pressure Analytical SEM with Oxford Microanalysis.

X-Ray Photoelectron Spectroscopy (XPS): XPS spectra were collected by the Kratos AXIS Supra+ equipped a variety of excitation sources

Fourier Transform Infrared Spectroscopy (FTIR): FTIR spectra were recorded at a range of 400 - 4000 cm^{-1} by using Jasco FTIR 4100 spectrometer.

Ultraviolet-visible Spectroscopy (UV-Vis): UV-Vis spectra were collected at 298 K by Shimadzu 1800 UV-visible spectrophotometer.

Dynamic Light Scattering (DLS) and Zeta Potential: The data of hydrodynamic size, polydispersity index (PDI) and zeta potential were measured by Litesizer LDS 500 instrument.

Inductively Coupled Plasma Optical Emission Spectroscopy (ICP-OES): ICP-OES data were collected by using Agilent 5900 ICP-OES instrument with an Agilent SPS 4 autosampler.

Confocal Laser Scanning Microscopy (CLSM): The images of CLSM were recorded by Zeiss LSM 880 inverted confocal microscope with AiryScan.

Magnetic Resonance Imaging (MRI): The images of MR were obtained by the MR scanner system (Bruker, BioSpec 94/30, USR).

S2. Synthesis, Drug Loading, and Postsynthetic Modification

S2.1 Synthesis and Characterisation of PEG-COOH

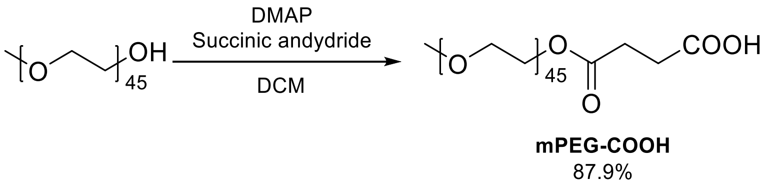

Scheme S1. Synthetic route of **mPEG-COOH**.

mPEG-COOH was synthesized according to the methods reported previously.[S1] Briefly, DMAP (610 mg, 5 mmol), succinic anhydride (500 mg, 5 mmol) and poly(ethylene glycol) methyl ether 2000 (mPEG2000, 1 g, 0.5 mmol) were added to 50 mL DCM in a round-bottom flask. The mixture was stirred at room temperature overnight. Then 50 mL DCM and 100 mL water were then added. After washing with 10% $NaHSO_4$ solution, the organic phase was dried, filtered, and concentrated to yield a white solid **mPEG-COOH** (920 mg, 87.9%). ^1H NMR (400 MHz, $CDCl_3$) δ (ppm): 4.26-4.24 (m, 2H), 3.81 (t, J = 4 Hz, 2H), 3.67-3.63 (m, 172H), 3.55-3.53 (m, 2H), 3.47-3.44 (m, 2H), 3.37 (s, 3H), 2.66-2.60 (m, 4H).

^1H NMR spectrum (Figure S1) is consistent with literature reports.[S1]

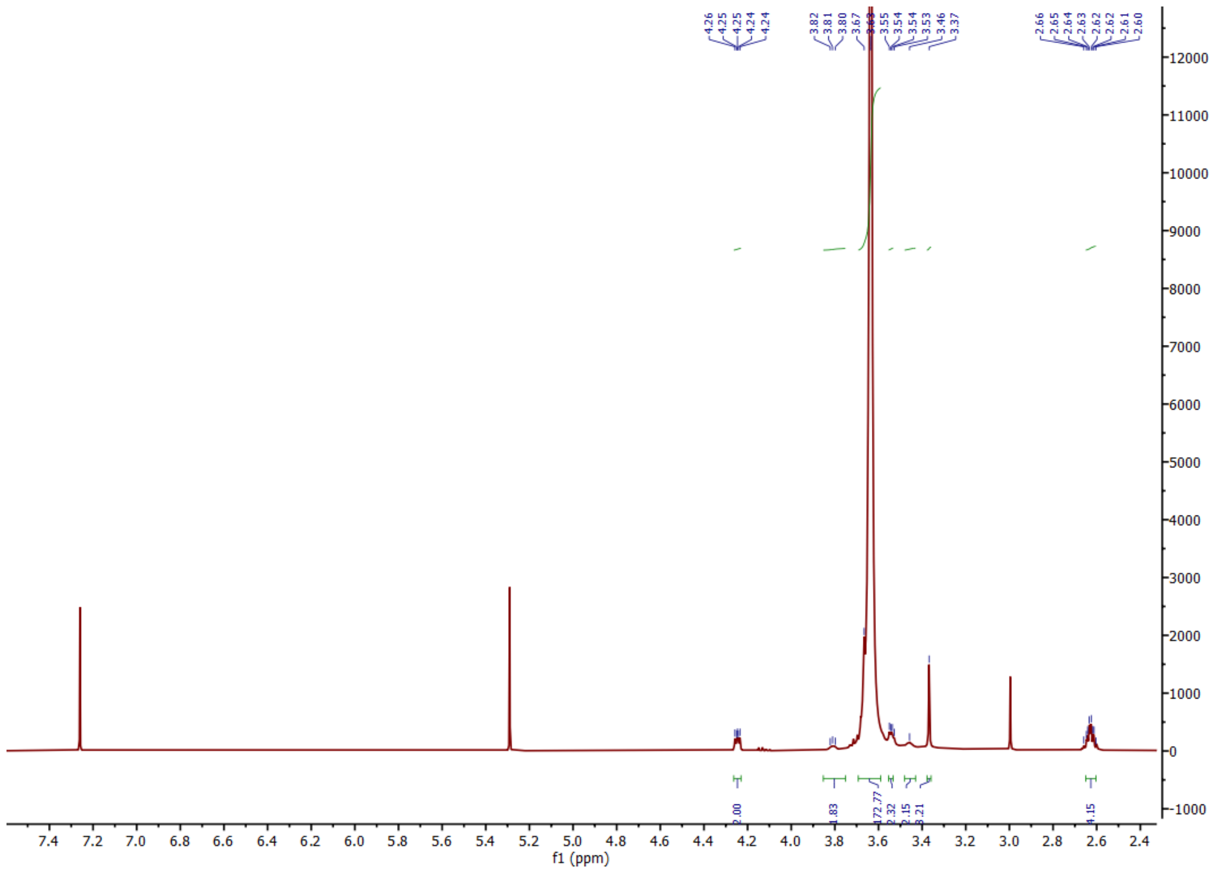

Figure S1. The ^{1}H NMR (400 MHz, $CDCl_3$) spectrum of **mPEG-COOH**.

S2.2 Synthesis of Fe-doped MOF-808

$ZrOCl_2·8H_2O$ (322 mg, 1.0 mmol), $FeCl_3$ (162 mg, 1.0 mmol), and benzene-1,3,5-tricarboxylic acid (210 mg, 1.0 mmol) were dissolved in 15 mL DMF and transferred to a glass jar. Acetic acid (15 mL) was then added to the mixture and then sonicated for 20 seconds, the mixture was kept in an oven at 403 K for 24 h. To remove the unreacted reagents, the mixture was washed with DMF (3 × 20 mL) and EtOH (3 × 20 mL) and dried in a desiccator overnight to obtain Fe-doped MOF-808 (MOF-808(Zr/Fe)). MOF-808(Zr/Fe) with different Fe loadings was synthesised by changing the ratio between $ZrOCl_2·8H_2O$ and $FeCl_3$. For Fe doping content measurement, MOF-808(Zr/Fe) samples with varying Fe doping content were digested in HNO_3 (2 mL) at 393 K. The resulting solution was diluted to 50 mL with water and analysed via ICP-OES to determine the Fe doping values.

Table S1. Fe content in Fe-doped MOFs with different ratios between $ZrOCl_2{\cdot}8H_2O$ and $FeCl_3$.

$FeCl_3/ZrOCl_2{\cdot}8H_2O$ ratios	Fe Content (w/w%)
0:10	0
1:9	0.61
2:8	1.52
3:7	2.14
4:6	2.87
5:5	3.95
6:4	3.98

The optimum combination of crystallinity and Fe loading was achieved with the 5:5 Zr:Fe synthetic ratio. This material was selected for further study, and is described here on as MOF-808(Zr/Fe).

S2.3 Activation of MOF-808(Zr/Fe)

MOF-808(Zr/Fe) (1.5 g) was dispersed in MeOH (600 mL) and stirred at room temperature for three days. Then the mixture was washed with MeOH (3 × 200 mL) and dried in a desiccator overnight to obtain activated MOF-808(Zr/Fe) (MOF-808(Zr/Fe) (act.)). The N_2 adsorption/desorption isotherms (77 K) and corresponding pore size distributions are given in Figure S2.

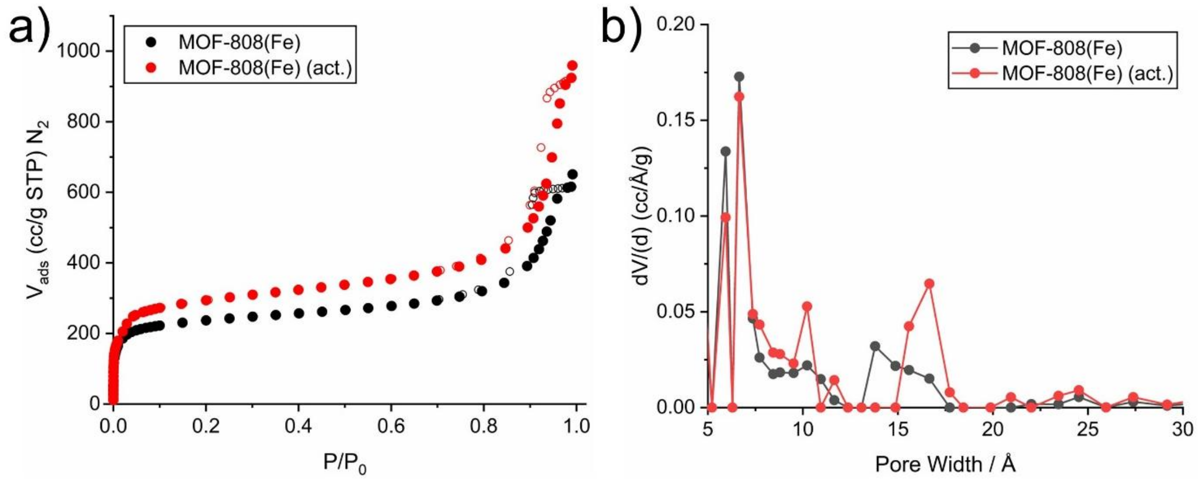

Figure S2. (a) N_2 adsorption/desorption isotherms (77 K filled symbols represent adsorption, empty symbols represent desorption) and (b) pore size distributions derived from the N_2 adsorption isotherms (QSDFT, slit cylinder/spherical pore) for MOF-808(Zr/Fe) before and post activation.

S2.4 Synthesis of Ultra-small Gold Nanoparticles Hybridized MOF-808(Zr/Fe)

MOF-808(Zr/Fe)-AuNP was obtained by using the incipient wetness impregnation method.[S2] Typically, MOF-808(Zr/Fe) (activated, 600 mg) was dispersed into 160 mL MeOH. After the $HAuCl_4$ solution (3.5 mL, 8 mg mL^{-1}) was added, the mixture was stirred at room temperature for 6 h under a nitrogen atmosphere. Next, $NaBH_4$ solution (800 µL, 0.1 M) was added, and the mixture was kept stirring for an additional hour. Finally, the mixture was washed with H_2O (2 × 100 mL) and EtOH (3 × 100 mL) and dried in a desiccator overnight to yield ultra-small gold nanoparticles hybridized MOF-808(Zr/Fe) (MOF-808(Zr/Fe)-AuNP).

S2.5 Drug Loading

Carboplatin (CA) was used as a model drug in this research. MOF-808(Zr/Fe)-AuNP (100 mg) was dispersed into 20 mL aqueous solution of CA (5 mg mL^{-1}) by sonication. The reaction mixture was stirred at room temperature for 24 h. To remove the unloaded

CA, the mixture was washed with water (3 × 20 mL) and EtOH (3 × 20 mL) and dried in a desiccator overnight to obtain carboplatin-loaded MOF-808(Zr/Fe)-AuNP (CA@MOF-808(Zr/Fe)-AuNP).

S2.6 Installation of Manganese Sites in CA@MOF-808(Zr/Fe)-AuNP

CA@MOF-808(Zr/Fe)-AuNP-Mn was synthesised according to the protocol reported previously by using the solvothermal deposition in MOFs method.[S3] CA@MOF-808(Zr/Fe)-AuNP (50 mg) was dispersed into DMF (12 mL) by sonicated. After adding $Mn(CH_3CO_2)_2{\cdot}4H_2O$ (95 mg), the mixture was stirred at room temperature for 24 h. To remove the unreacted reagent, the mixture was washed with DMF (3 × 10 mL) and EtOH (3 × 10 mL) and dried in a desiccator overnight to get Mn-installed CA@MOF-808(Zr/Fe)-AuNP (CA@MOF-808(Zr/Fe)-AuNP-Mn).

S2.7 Surface PEGylation of CA@MOF-808(Zr/Fe)-AuNP-Mn

CA@MOF-808(Zr/Fe)-AuNP-Mn (100 mg) was dispersed into 50 mL EtOH solution of mPEG-COOH (2 mg mL^{-1}) by sonication. Then, the mixture was stirred at room temperature for 24 h. To remove the unreacted mPEG-COOH, the mixture was washed with EtOH (3 × 30 mL) and dried in a desiccator overnight to obtain PEGylated CA@MOF-808(Zr/Fe)-AuNP-Mn (CA@MOF-808(Zr/Fe)-AuNP-Mn-PEG).

S3. Characterisation

S3.1. Additional Sample Characterisation Methods

S3.1.1. Drug Loading Capacity

CA@MOF-808(Zr/Fe)-AuNP-Mn-PEG (2.5 mg) were digested in aqua regia (2 mL) at 393 K. The resultant solution was diluted to 50 mL with water and then analysed by ICP-OES (standard solutions were obtained from Element Materials Technology Laboratory Solutions UK Ltd.). The content of CA was calculated from platinum results according to the molecular weight of CA.

The drug loading capacity was calculated by using the following formula:

$$\text{Loading capacity of CA (\%)} = \frac{M_{CA}}{M_{MOF}} \times 100$$

Where M_{CA} is the mass of loaded CA and M_{MOF} is the mass of CA@MOF-808(Zr/Fe)-AuNP-Mn-PEG.

S3.1.2. Phosphate-Responsive Drug Release Profile

CA@MOF-808(Zr/Fe)-AuNP-Mn-PEG (4 mg) was dispersed in 20 mL of phosphate buffer saline (pH = 7.4) with different concentrations of phosphate (10 mM or 2 mM). At each predetermined interval (0.25, 0.5, 1, 2, 4, 8, 12, 24, 48 h), 2 mL of portion was taken out and centrifuged to collect the supernatant. The released platinum in supernatant was measured by ICP-OES.

S3.1.3. Hydroxyl Radical (•OH) Generation

The·•OH generation was measured by TMB assay in different groups (MOF-808(Zr/Fe)-PEG ± H_2O_2 and MOF-808(Zr/Fe)-AuNP-Mn-PEG ± H_2O_2) dispersed in

PBS (TMB: 50 μg mL^{-1}, H_2O_2: 10 mM, MOFs: 250 μg/mL, pH = 7.4 and 6.0). After 30 min reaction, the mixture was centrifuged and the supernatant was measured by UV-Vis spectrophotometer (300-800 nm).

For ESR measurements, DMPO was used as •OH trap. Briefly, 20 μL DMPO was added in MOF-808(Zr/Fe)-AuNP-Mn-PEG solution (200 μg mL^{-1}, 2 mL) containing 10 mM H_2O_2, and then the mixture was run by ESR equipment.

S3.1.4. *In Vitro* Magnetic Resonance Imaging (MRI)

CA@MOF-808(Zr/Fe)-AuNP-Mn-PEG with various concentrations (0, 0.25, 0.5 and 1 mg mL^{-1}, corresponding to Mn concentration: 0, 0.16, 0.32 and 0.63 mM) was dispersed in PBS with different conditions (pH = 5.0, GSH: 0 mM; pH = 5.0, GSH: 10 mM; pH = 7.4, GSH: 0 mM). Subsequently, the resultant mixture was reacted for 1 h at 310 K and then scanned under an MR scanner system to obtain T_1-weighted MRI.

S3.2. Additional Characterisation of CA@MOF-808(Zr/Fe)-AuNP-Mn-PEG and Precursors

Exemplar characterisation from each synthetic step in the preparation of CA@MOF-808(Zr/Fe)-AuNP-Mn-PEG is provided in Figure 2 in the manuscript. Additional characterisation for CA@MOF-808(Zr/Fe)-AuNP-Mn-PEG follow.

Scanning electron micrographs and corresponding particle size distributions are given in Figure S3.

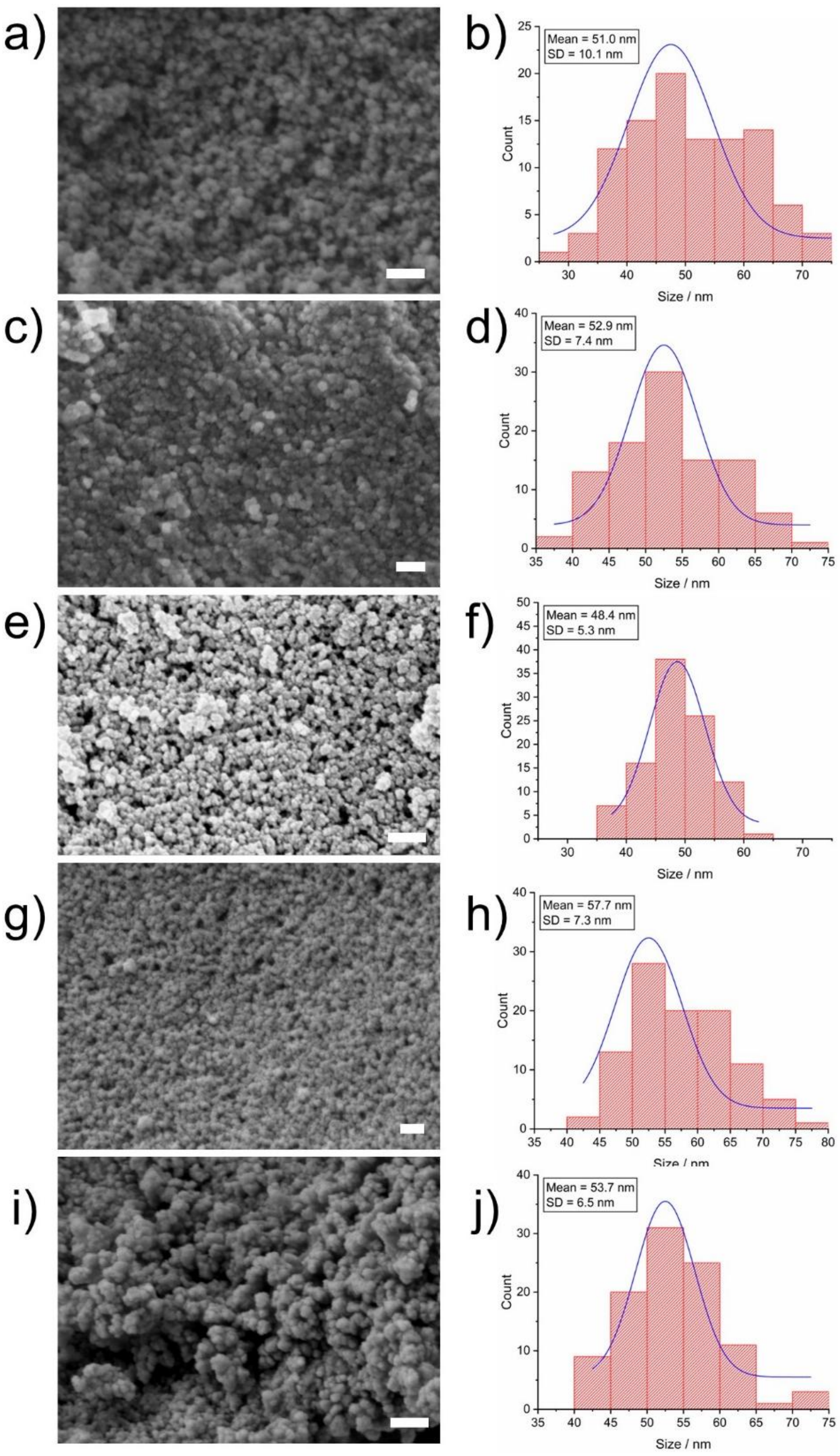

Figure S3. The SEM images and particles size distribution histograms of (a, b) MOF-808(Zr/Fe), (c, d) MOF-808(Zr/Fe)-AuNP, (e, f) CA@MOF-808(Zr/Fe)-AuNP, (g, h) CA@MOF-808(Zr/Fe)-AuNP-Mn and (i, j) CA@MOF-808(Zr/Fe)-AuNP-Mn-PEG. The scale bar in SEM images is 200 nm.

FTIR spectra are given in Figure S4.

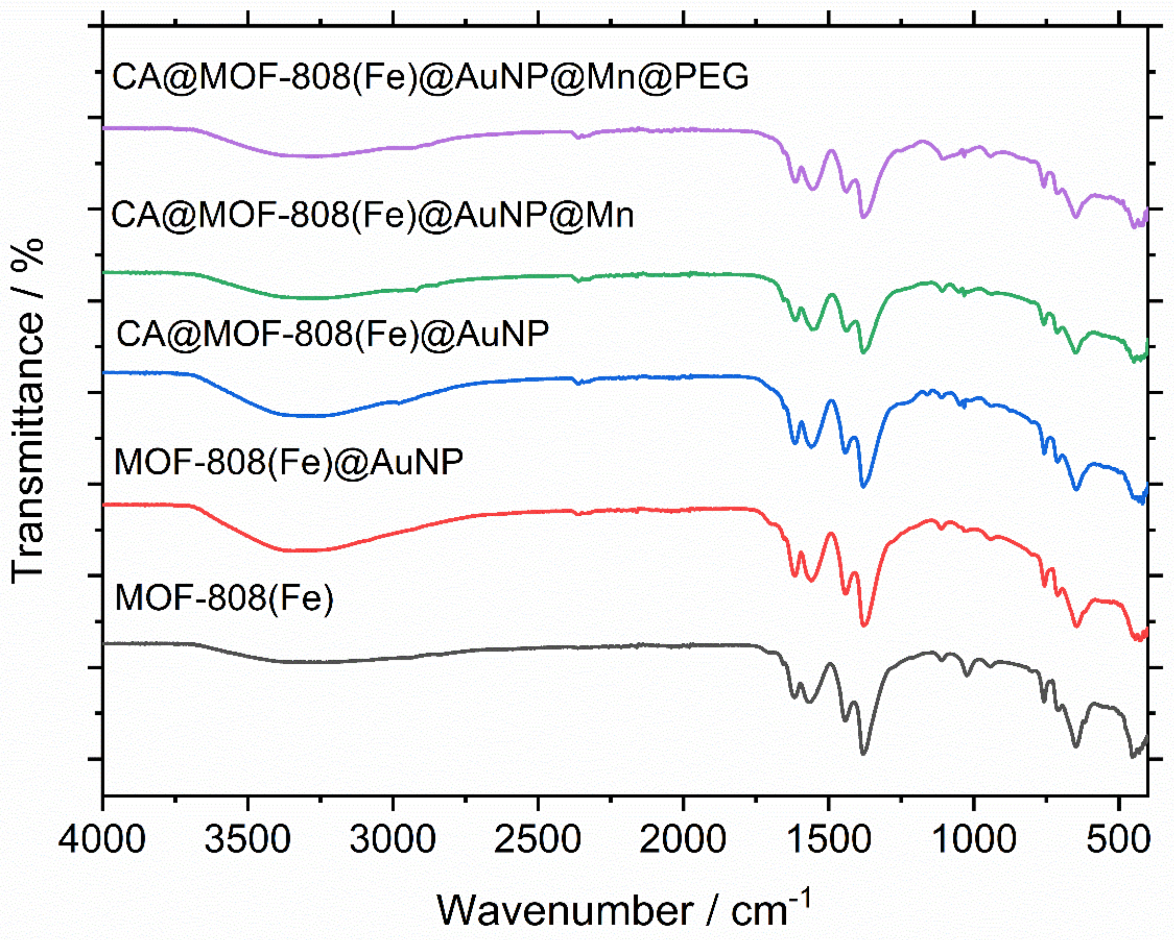

Figure S4. FT IR spectra of MOF-808(Zr/Fe) at different stages of drug loading and surface modification.

BET areas and pore volumes derived from the N_2 adsorption desorption isotherms in Figure 2g of the main manuscript are given in Table S2, while the metal content of the varying components of CA@MOF-808(Zr/Fe)-AuNP-Mn-PEG are given in Table S3.

Table S2. Table of BET areas and porosities of MOF-808(Zr/Fe) at different stages of drug loading and surface modification.

Samples	BET Surface Area (m^2/g)	Pore Volume (cc/g)
MOF-808(Fe)	871.91	0.3573
MOF-808(Fe) (act.)	1078.23	0.4394
MOF-808(Fe)@AuNP	968.12	0.3972
CA@MOF-808(Fe)@AuNP	759.62	0.3098
CA@MOF-808(Fe)@AuNP@Mn	568.41	0.2318
CA@MOF-808(Fe)@AuNP@Mn@PEG	498.90	0.2031

Table S3. Different metal content (w/w%) in CA@MOF-808(Zr/Fe)-AuNP-Mn-PEG by ICP-OES.

Metal Element	w/w%
Zr	19.05
Fe	3.44
Mn	3.5
Au	1.6
Pt	3.21

XPS spectra of selected O, C and Zr regions for MOF-808(Zr/Fe)-AuNP-Mn-PEG are provided in Figure S5, and complement those in the main manuscript, Figures 3a–3d.

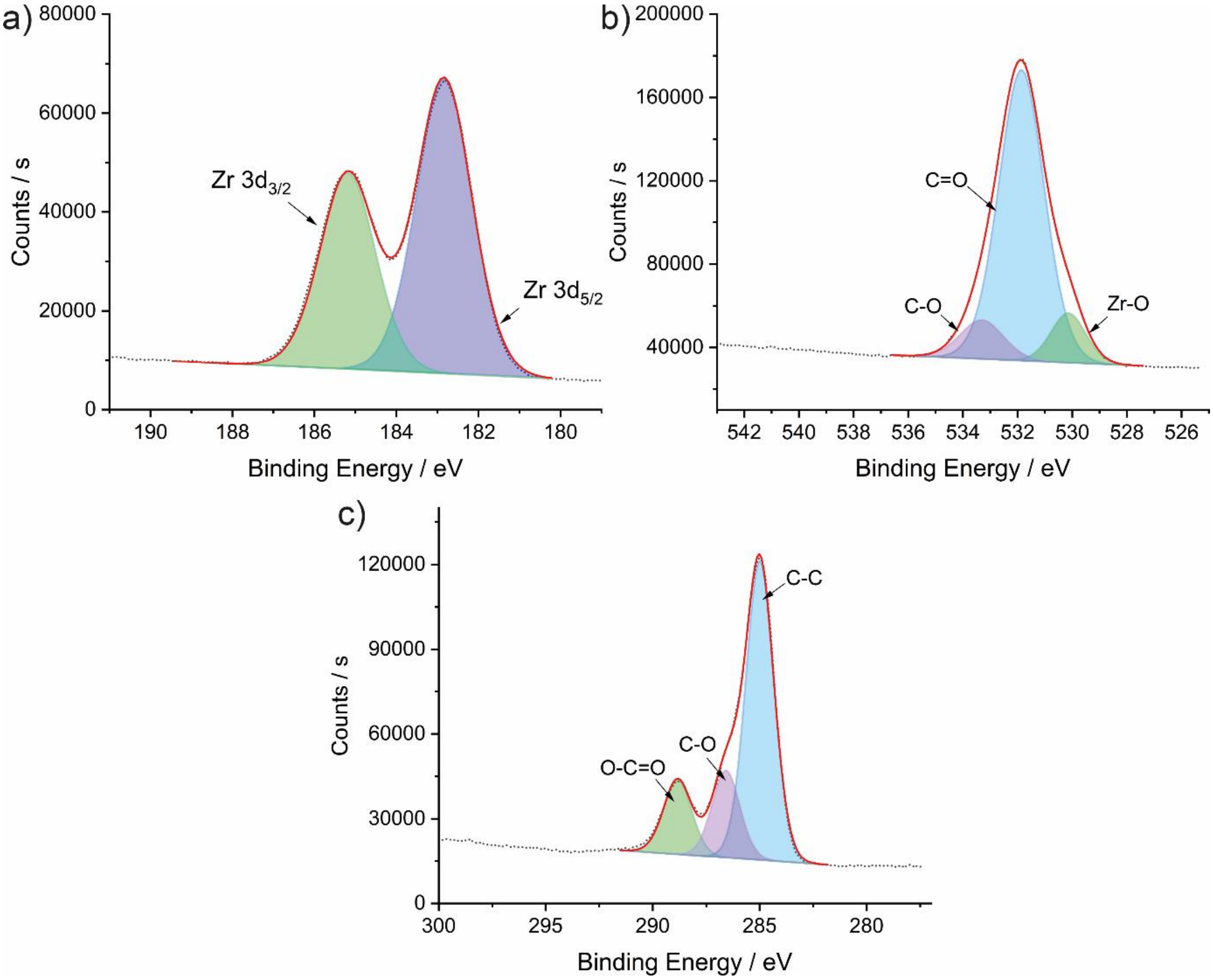

Figure S5. XPS spectra of a) Zr 3d, b) O 1s, and c) C 1s orbitals of CA@MOF-808(Zr/Fe)-AuNP-Mn-PEG.

The EDS spectrum of CA@MOF-808(Zr/Fe)-AuNP-Mn-PEG (Figure S6) complements the elemental mapping in Figure 3e of the main manuscript.

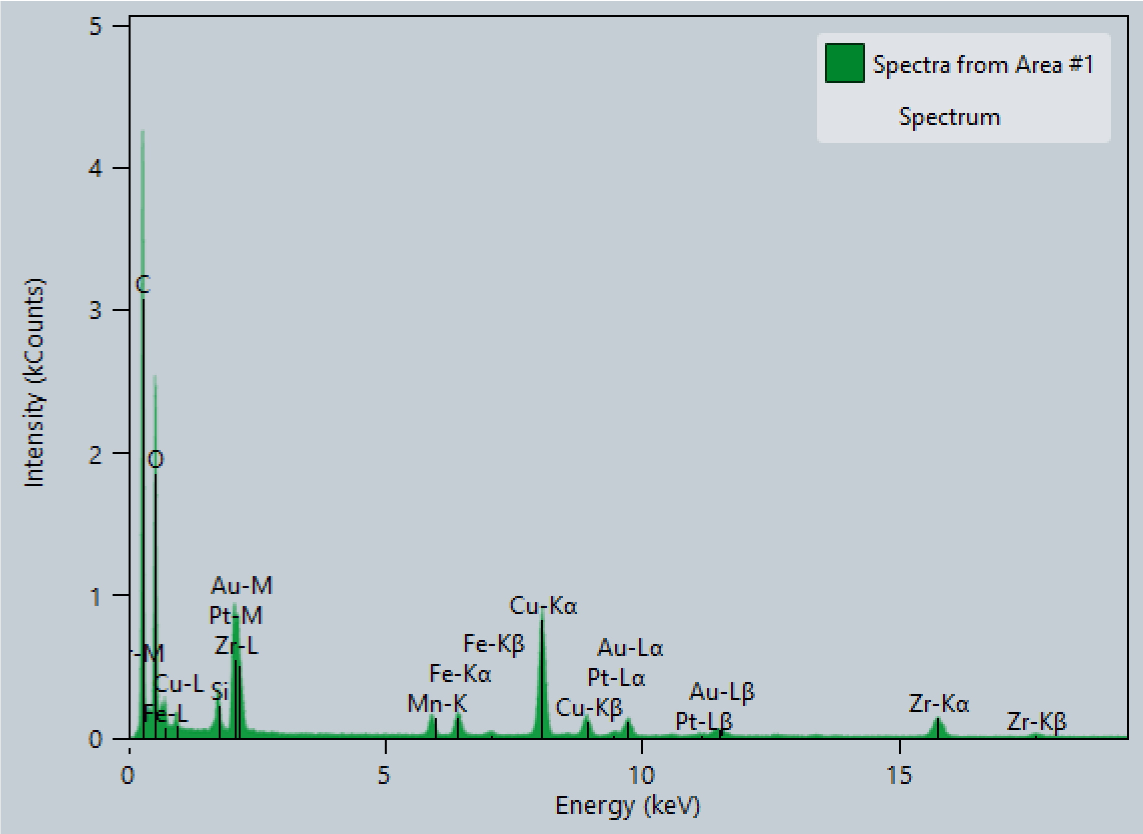

Figure S6. EDS spectrum of CA@MOF-808(Zr/Fe)-AuNP-Mn-PEG.

S3.3. Characterisation of Control Samples

The control samples (MOF-808(Zr/Fe)-PEG, MOF-808(Zr/Fe)-AuNP-PEG, MOF-808(Zr/Fe)-Mn-PEG, MOF-808(Zr/Fe)-AuNP-Mn-PEG, CA@MOF-808(Zr/Fe)-PEG, CA@MOF-808(Zr/Fe)-AuNP-PEG, and CA@MOF-808(Zr/Fe)-Mn-PEG.) were synthesised following the protocols described in Section S2. Powder X-ray diffractograms are provided in Figures S7 and S8, thermogravimetric analyses in Figures S9 and S10, SEM analysis in Figure S11, and metal ion contents from ICP-OES in Table S4.

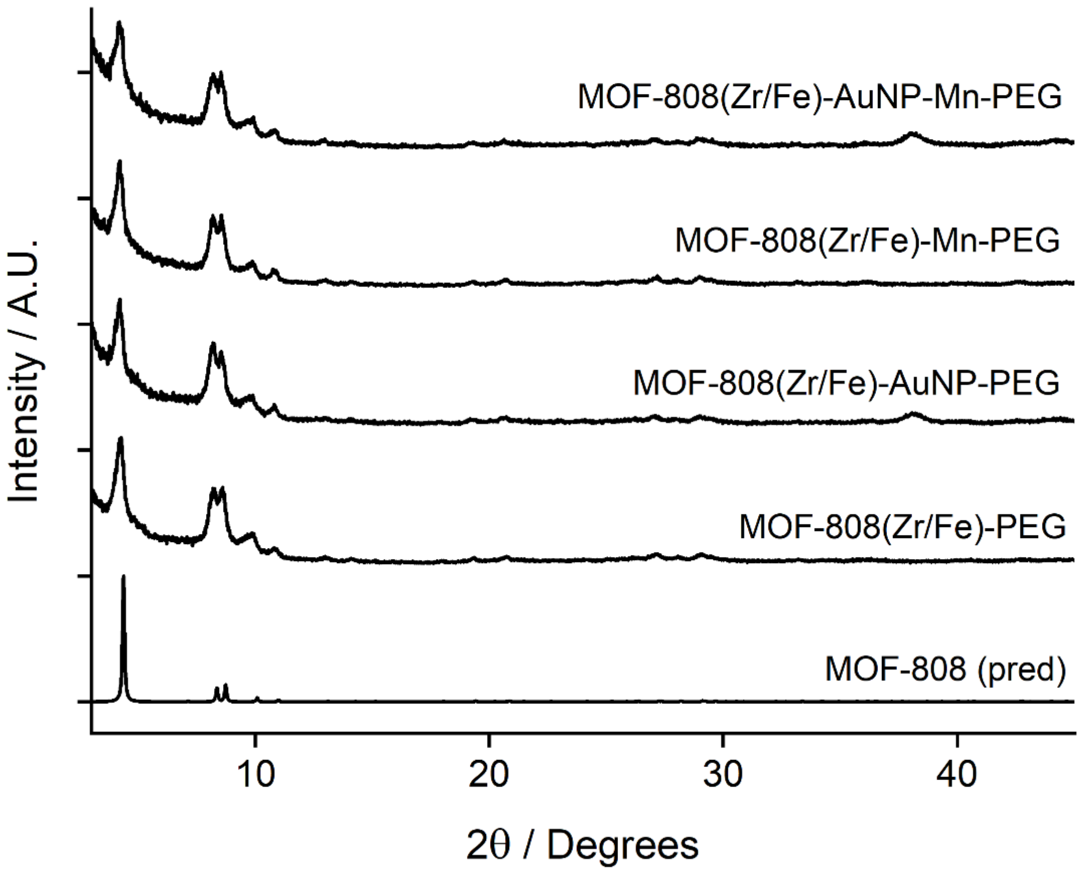

Figure S7. Stacked powder X-ray diffractograms of modified MOF-808(Zr/Fe) samples without CA loading compared to the pattern predicted for MOF-808.

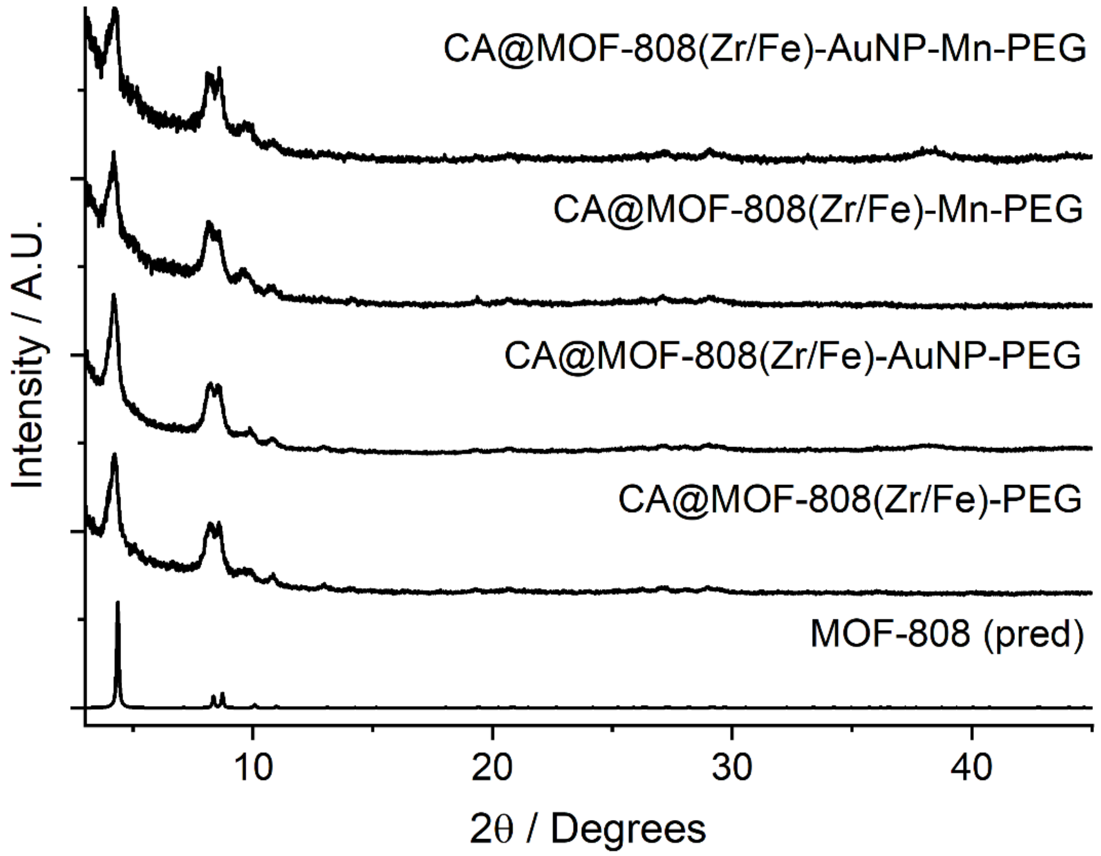

Figure S8. Stacked powder X-ray diffractograms of modified MOF-808(Zr/Fe) samples with CA loading compared to the pattern predicted for MOF-808.

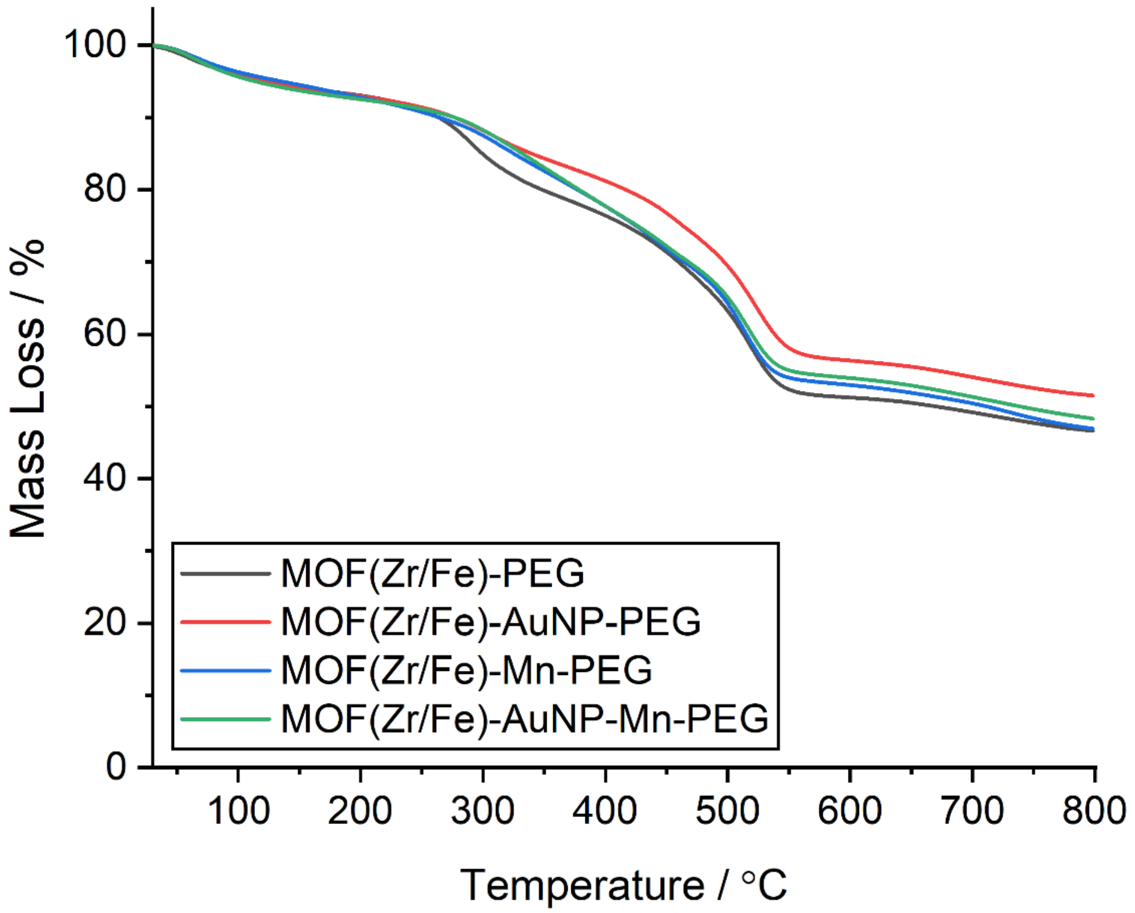

Figure S9. Thermogravimetric analysis traces for the sequentially modified MOF-808(Zr/Fe) samples without CA loading.

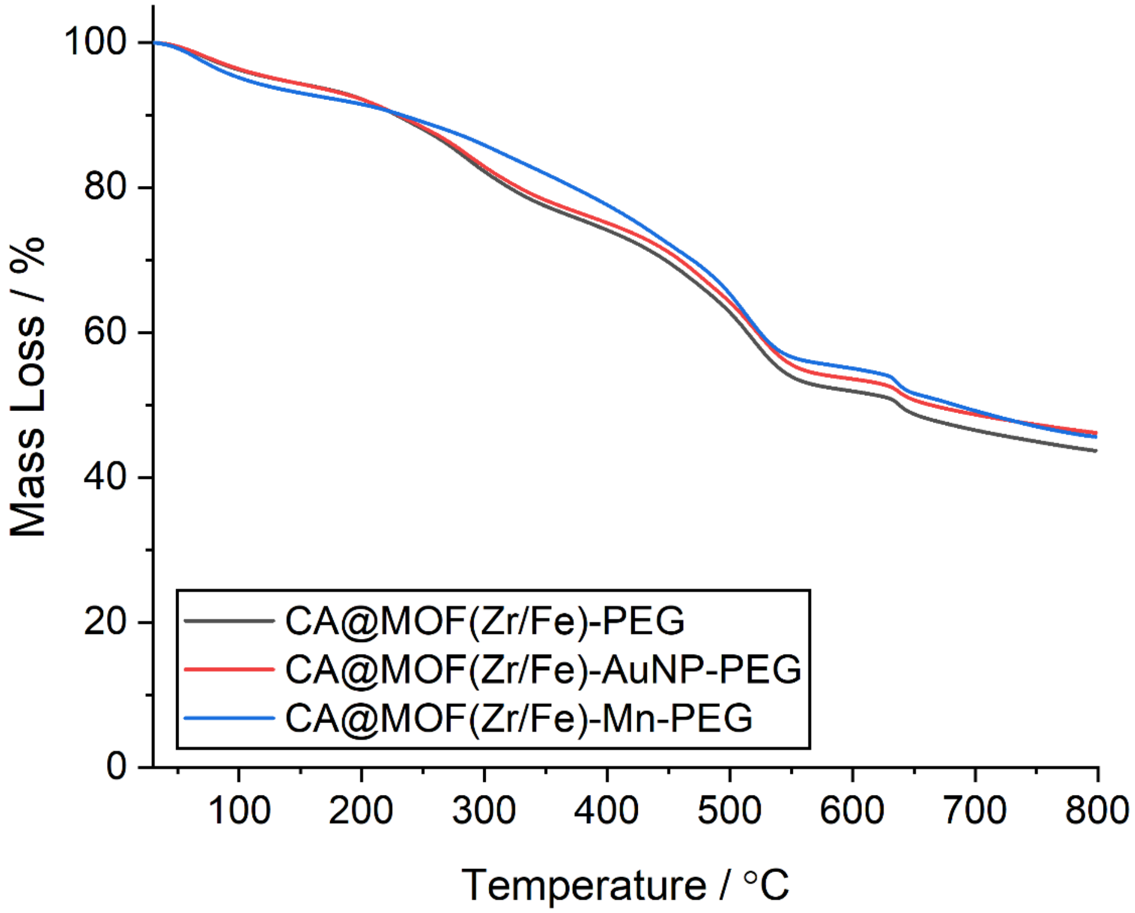

Figure S10. Thermogravimetric analysis traces for the sequentially modified MOF-808(Zr/Fe) samples with CA loading.

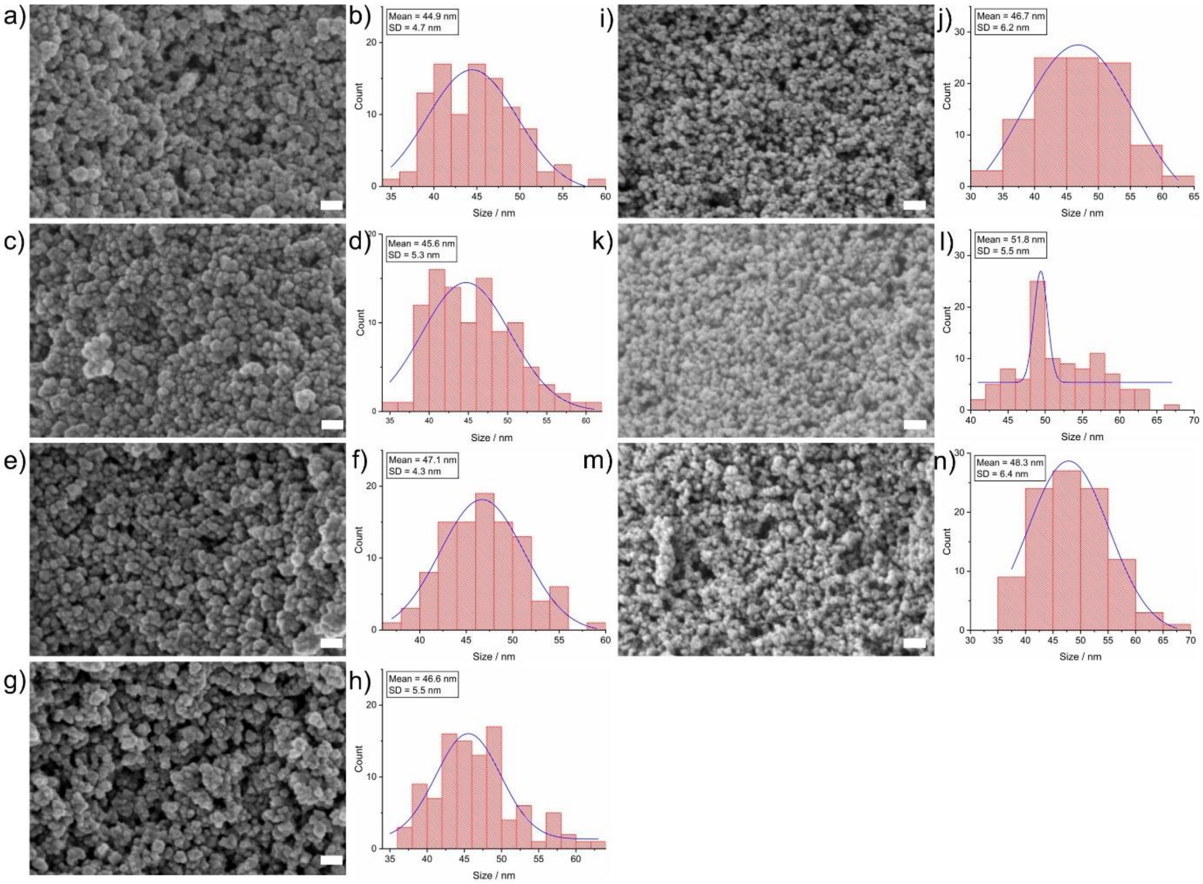

Figure S11. The SEM images and particles size distribution histograms of (a, b) MOF-808(Zr/Fe)-PEG, (c, d) MOF-808(Zr/Fe)-AuNP-PEG, (e, f) MOF-808(Zr/Fe)-Mn-PEG, (g, h) MOF-808(Zr/Fe)-AuNP-Mn-PEG, (i, j) CA@MOF-808(Zr/Fe)-PEG, (k, l) CA@MOF-808(Zr/Fe)-AuNP-PEG, and (m, n) CA@MOF-808(Zr/Fe)-Mn-PEG. The scale bar in SEM images is 100 nm.

Table S4. Different metal content (wt%) in varying modified MOF-808(Zr/Fe) samples by ICP-OES.

Samples	Au (wt%)	Fe (wt%)	Mn (wt%)	Pt (wt%)	Zr (wt%)
MOF-808(Zr/Fe)-PEG	/	4.3	/	/	15.4
MOF-808(Zr/Fe)-AuNP-PEG	2.5	4.2	/	/	14.2
MOF-808(Zr/Fe)-Mn-PEG	/	3.6	3.4	/	12.0
MOF-808(Zr/Fe)-AuNP-Mn-PEG	2.5	4	3.6	/	12.5
CA@MOF-808(Zr/Fe)-PEG	/	3.7	/	3.6	10.4
CA@MOF-808(Zr/Fe)-AuNP-PEG	1.8	3.9	/	3.9	10.0
CA@MOF-808(Zr/Fe)-Mn-PEG	/	3.6	5.6	2.6	10.7
CA@MOF-808(Zr/Fe)-AuNP-Mn-PEG	1.6	3.4	3.5	3.2	19.0

S4. *In Vitro* Experiments

S4.1. Cell Culture

HepG2 (human hepatoma carcinoma cell line) and HEK 293 (human embryonic kidney 293 cell line) were cultured in Dulbecco's Modified Eagle Medium supplemented with 10% fetal bovine serum, 1% penicillin-streptomycin and 1% L-glutamine. Cells were incubated in a humidified atmosphere at 310 K in 5% CO_2.

S4.2. Calcein Loading

Calcein loading of MOF-808(Zr/Fe)-AuNP-Mn-PEG: Calcein (50 mg, Cal) was dissolved in MeOH (50 mL), and then MOF-808(Zr/Fe)-AuNP (50 mg) was added in and dispersed by sonication. The reaction solution was stirred in a dark condition for 24 h at room temperature. Then, the mixture was washed with MeOH (3 × 50 mL) and dried in a desiccator overnight to obtain Cal@MOF-808(Zr/Fe)-AuNP. After manganese ion immobilisation and PEGylation, which were carried out by the same processes as described in Sections S2.6 and S2.7, Cal@MOF-808(Zr/Fe)-AuNP-Mn-PEG was obtained. Cal content was measured by UV-Vis spectroscopy of a PBS-digested sample. Cal@MOF-808(Zr/Fe)-AuNP-Mn-PEG (2.4 mg) was dispersed in PBS (1X, 10 mL) and stirred for one week under dark conditions. The sample solution was then centrifuged and the supernatant was measured by UV-Vis spectroscopy, allowing the mass of loaded calcein to be calculated by calcein absorbance against the calibration curve (Figure S12).

The cal loading capacity was calculated by using the following formula:

$$\text{Loading capacity of Cal (\%)} = \frac{M_{Cal}}{M_{MOF}} \times 100$$

Where M_{Cal} is the mass of loaded Cal and M_{MOF} is the mass of Cal@MOF-808(Zr/Fe)-AuNP-Mn-PEG.

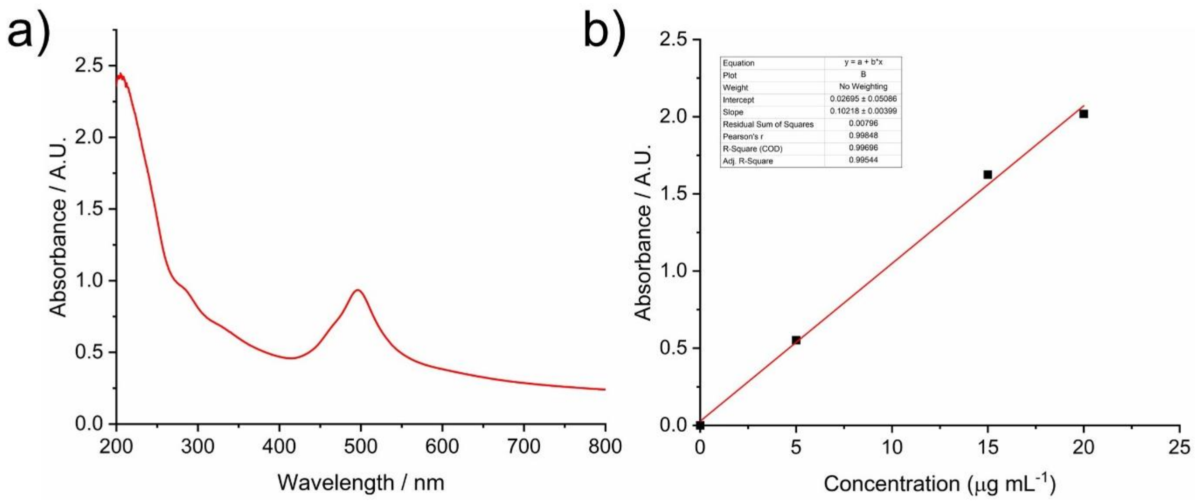

Figure S12. (a) UV-Vis spectrum of Cal@MOF-808(Zr/Fe)-AuNP-Mn-PEG. (b) Standard curve of calcein in PBS (pH 7.4). Calcein shows a strong absorption peak at 495 nm.

S4.3. ICP-OES for Quantitive Cell Uptake

HEK 293 and HepG2 cells were seeded into 6-well plates (1.5×10^6 cells/well) and incubated for 1 day, respectively. The old medium was replaced by CA@MOF-808(Zr/Fe)-AuNP-Mn-PEG-containing fresh medium (50 μg mL^{-1}) and the cells were further incubated for 1, 2, 4 and 8 h. Then, the cells were gently washed with PBS three times to remove non-uptaken MOF, and then detached by trypsin-EDTA (185 μL) and resuspended in fresh medium (815 μL). Finally, the cells were counted, centrifuged, and digested by aqua regia for ICP-OES analysis.

S4.4. CLSM for Qualitative Cell Uptake

Calcein was utilized as the tracer agent. Typically, HepG2 cells were seeded into Nunc™ Lab-Tek™ II 8-wells chamber slide (2×10^4 cells/well) and incubated for 1 day. Then the old medium was replaced by Cal@MOF-808(Zr/Fe)-AuNP-Mn-PEG-containing fresh medium (50 μg/mL) and the cells were incubated for 6 h. After removing non-uptaken MOF by DPBS washing, the cells were stained by CellMask™ Deep Red Plasma Membrane Stains and fixed by 4% paraformaldehyde. Then, the

chamber was removed and one drop of ProLong™ Glass Antifade Mountant was added to each well for CLSM imaging.

S4.5. Intracellular Hydroxyl Radical Generation

HepG2 cells were seeded at a density of 2 × 10^4 cells/well into Nunc™ Lab-Tek™ II 8-well chamber slide and incubated for 24 h. The old medium was then replaced with a fresh sample-containing medium (CA@MOF-808(Zr/Fe)-PEG, CA@MOF-808(Zr/Fe)-AuNP-PEG, CA@MOF-808(Zr/Fe)-Mn-PEG or CA@MOF-808(Zr/Fe)-AuNP-Mn-PEG; concentration: 200 μg mL^{-1}) and the HepG2 cells were further incubated for 24 h. After being washed with DPBS, the HepG2 cells were stained with a DCFH-DA working solution (20 μM) for 45 min and then washed with DPBS for three times again. Subsequently, the chamber was removed, and a drop of ProLong™ Glass Antifade Mountant was added to each well. Finally, a coverslip was placed on the slide, which was then imaged by using CLSM.

S4.6. Mitochondrial Membrane Potential Change

HepG2 cells were seeded at a density of 8 × 10^3 cells/well into a 96-well plate and incubated for 24 h. The old medium was then replaced with a fresh medium containing either MOF-808(Zr/Fe)-based nanocomposite samples (CA@MOF-808(Zr/Fe)-PEG, CA@MOF-808(Zr/Fe)-AuNP-PEG, CA@MOF-808(Zr/Fe)-Mn-PEG or CA@MOF-808(Zr/Fe)-AuNP-Mn-PEG; concentration: 200 μg mL^{-1}) or the corresponding concentration of free CA (12 μg mL^{-1}), and the HepG2 cells were further incubated for 24 h. After that, 50 μL of JC-10 dye-loading solution was added to each well and the HepG2 cells were incubated for an additional hour. Subsequently, 50 μL of JC-10 assay buffer B was added to each well. Finally, the fluorescence intensity ratio analysis was measured by using CLARIOstar microplate reader ($\lambda_{ex/em}$ = 490 nm/525 nm, cut off at 515 nm; and $\lambda_{ex/em}$ = 540 nm/590 nm, cut off at 570 nm).

S4.7. Intracellular ATP Content Detection

HepG2 cells were seeded at a density of 2.5×10^4 cells/well into a 96-well plate and incubated for 24 h. The old medium was then replaced with a fresh medium containing either MOF-808(Zr/Fe)-based nanocomposite samples (CA@MOF-808(Zr/Fe)-PEG, CA@MOF-808(Zr/Fe)-AuNP-PEG, CA@MOF-808(Zr/Fe)-Mn-PEG or CA@MOF-808(Zr/Fe)-AuNP-Mn-PEG; concentration: 200 μg mL^{-1}) or the corresponding concentration of free CA (12 μg mL^{-1}), and the HepG2 cells were further incubated for 24 h. After that, 50 μL of detergent was added to each well, the 96-well plate was sealed and shaken in an orbital shaker for 5 min. Subsequently, 50 μL of Substrate Solution was added to each well, the 96-well plate was sealed and shaken in an orbital shaker for 5 min again. Next, the 96-well plate was kept for 10 min at room temperature under dark conditions. Finally, the luminescence intensities were measured by using CLARIOstar microplate reader.

S4.8. *In Vitro* Cytotoxicity Assay

HepG2 cells were seeded in 96-well plates (8×10^3 cells/well). After 24 h incubation, fresh medium was added, which contained different concentrations of MOF-808(Zr/Fe)-PEG, MOF-808(Zr/Fe)-AuNP-PEG, MOF-808(Zr/Fe)-Mn-PEG, MOF-808(Zr/Fe)-AuNP-Mn-PEG, CA@MOF-808(Zr/Fe)-PEG, CA@MOF-808(Zr/Fe)-AuNP-PEG, CA@MOF-808(Zr/Fe)-Mn-PEG, CA@MOF-808(Zr/Fe)-AuNP-Mn-PEG (10, 25, 50, 100, 150, 200 μg mL^{-1}), or corresponding concentrations of free CA (0.6, 1.5, 3, 6, 9, 12 μg mL^{-1}) and incubated for further 24, 48 and 72 h. Next, fresh medium containing 10% AlamarBlue™ Cell Viability Reagent was and the cell viabilities were measured by using CLARIOstar microplate reader ($\lambda_{ex/em}$ = 557 nm/593 nm).

Cell viabilities of the CA-free samples towards HepG2 are plotted in Figure S13, to complement those of the CA-loaded analogues in Figures 6d–6f of the main manuscript. An alternative plot of these data, normalised to CA content, is provided in Figure S14. Viability data of HEK-293 cells towards CA@MOF-808(Zr/Fe)-AuNP-Mn-

PEG are presented in Figure S15. IC_{50} values derived from these data are provided in Table S5.

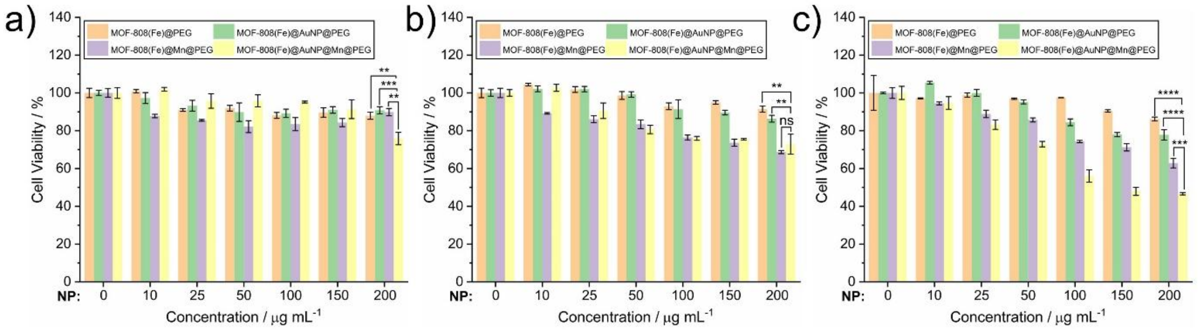

Figure S13. Cell viabilities of HepG2 cells incubated with different concentrations of MOF-808(Zr/Fe)-PEG, MOF-808(Zr/Fe)-AuNP-PEG, MOF-808(Zr/Fe)-Mn-PEG, and MOF-808(Zr/Fe)-AuNP-Mn-PEG for (a) 24 h, (b) 48 h and (c) 72 h. Data shown are mean of three biological replicates, error bars denote standard deviations. Statistical significance of 200 μg mL^{-1} dose calculated by one-way analysis of variance (ANOVA): $^{**}p < 0.01$, $^{***}p < 0.001$, $^{****}p < 0.0001$, and ns means not significant.

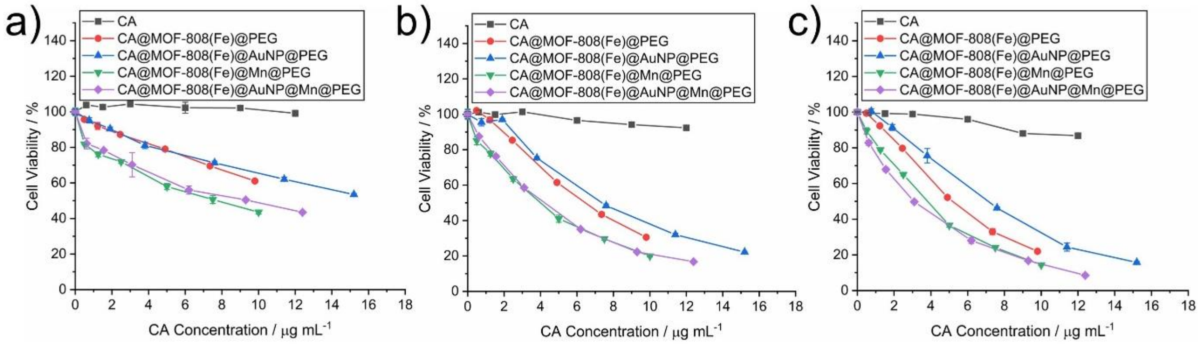

Figure S14. Cell viabilities of HepG2 cells incubated with CA, CA@MOF-808(Zr/Fe)-PEG, CA@MOF-808(Zr/Fe)-AuNP-PEG, CA@MOF-808(Zr/Fe)-Mn-PEG, and CA@MOF-808(Zr/Fe)-AuNP-Mn-PEG for (a) 24, (b) 48 and (c) 72 h, plotted against CA concentration. Represented as bar graphs in main manuscript, Figure 6d–6f. Data used to calculate IC_{50} values in Table S5.

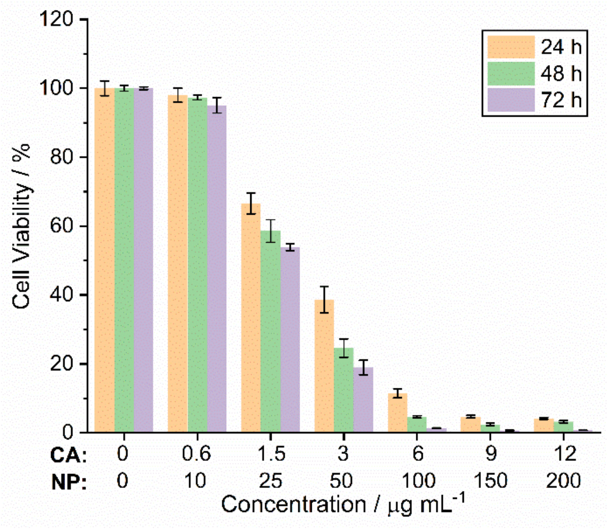

Figure S15. Cell viabilities of HEK 293 cells incubated with different concentrations of CA@MOF-808(Zr/Fe)-AuNP-Mn-PEG for (a) 24 h, (b) 48 h and (c) 72 h. Data shown are mean of three biological replicates, error bars denote standard deviations.

Table S5. Table of IC_{50} of different samples in cancer cell line (HepG2) and normal cell line (HEK 293). Unreported values are the values that could not be determined in the used sample concentration range, n = 3.

Samples	Cell lines	IC_{50} (μg mL^{-1})
CA@MOF-808(Fe)@AuNP@Mn@PEG	HEK 293	27.14 ± 1.01
CA	HepG2	/
CA@MOF-808(Fe)@PEG	HepG2	103.4 ± 1.00
CA@MOF-808(Fe)@AuNP@PEG	HepG2	88.96 ± 1.02
CA@MOF-808(Fe)@Mn@PEG	HepG2	68.68 ± 1.03
CA@MOF-808(Fe)@AuNP@Mn@PEG	HepG2	44.74 ± 1.03
MOF-808(Fe)@PEG	HepG2	/
MOF-808(Fe)@AuNP@PEG	HepG2	/
MOF-808(Fe)@Mn@PEG	HepG2	/
MOF-808(Fe)@AuNP@Mn@PEG	HepG2	147.4 ± 1.05

S4.9. Tumourigenic Sphere Formation Assay and Viability Assay

For the tumourigenic sphere formation assay, we used a special tumoursphere medium for cell culture, where DMEM medium (100 mL) contains penicillin-streptomycin (1 mL), L-glutamine (1 mL), B-27 supplement (2 mL) and recombinant human EGF lyophilized (0.1 mL). HepG2 cells (2500 cells/well) were seeded in Corning™ 96-well clear ultralow-attachment microplates separately and incubated with different concentrations of MOF-808(Zr/Fe)-PEG, MOF-808(Zr/Fe)-AuNP-PEG, MOF-808(Zr/Fe)-Mn-PEG, MOF-808(Zr/Fe)-AuNP-Mn-PEG, CA@MOF-808(Zr/Fe)-PEG, CA@MOF-808(Zr/Fe)-AuNP-PEG, CA@MOF-808(Zr/Fe)-Mn-PEG, CA@MOF-808(Zr/Fe)-AuNP-Mn-PEG (6.25, 12.5, 25, 50 μg mL^{-1}) or corresponding concentrations of free CA (0.375, 0.75, 1.5, 3, 6 μg mL^{-1}). After 5 days of incubation, tumour spheroids were photographed by microscope. Then, AlamarBlue™ Cell Viability Reagent (10%) was added and the cell viabilities were measured by using CLARIOstar microplate reader ($\lambda_{ex/em}$ = 557 nm/593 nm).

The cell viabilies are plotted as bar graphs in Figure 7b of the main manuscript, with a complementary line graph normalised to CA content provided in Figure S16. Control data for the incubation of HepG2 spheroids with free CA is shown in Figure S17, and derived IC_{50} values are provided in Table S6.

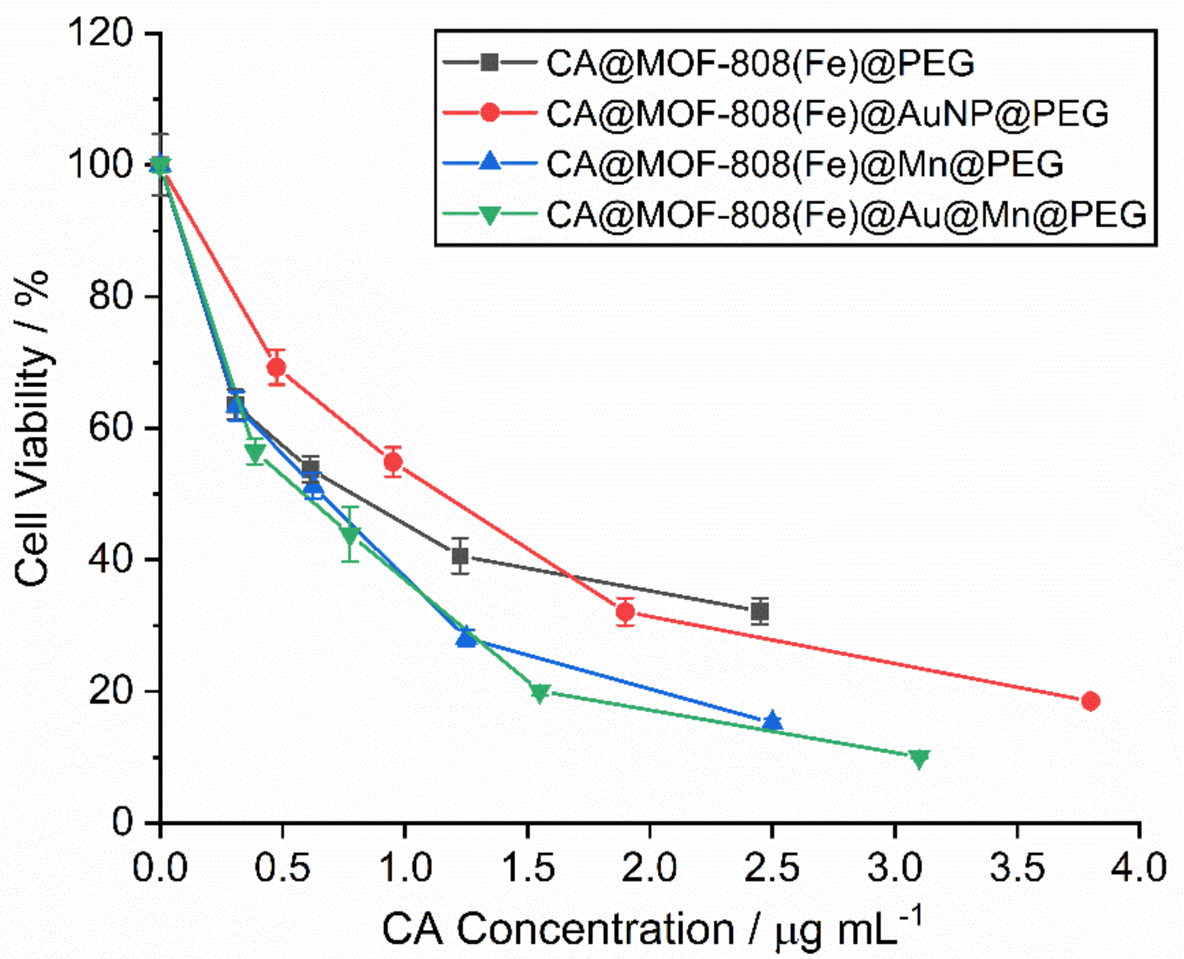

Figure S16. Cell viabilities of HepG2 spheroids incubated with CA@MOF-808(Zr/Fe)-PEG, CA@MOF-808(Zr/Fe)-AuNP-PEG, CA@MOF-808(Zr/Fe)-Mn-PEG, and CA@MOF-808(Zr/Fe)-AuNP-Mn-PEG for 5 days, plotted against CA concentration. Represented as bar graphs in main manuscript, Figure 7b. Data used to calculate IC_{50} values in Table S6.

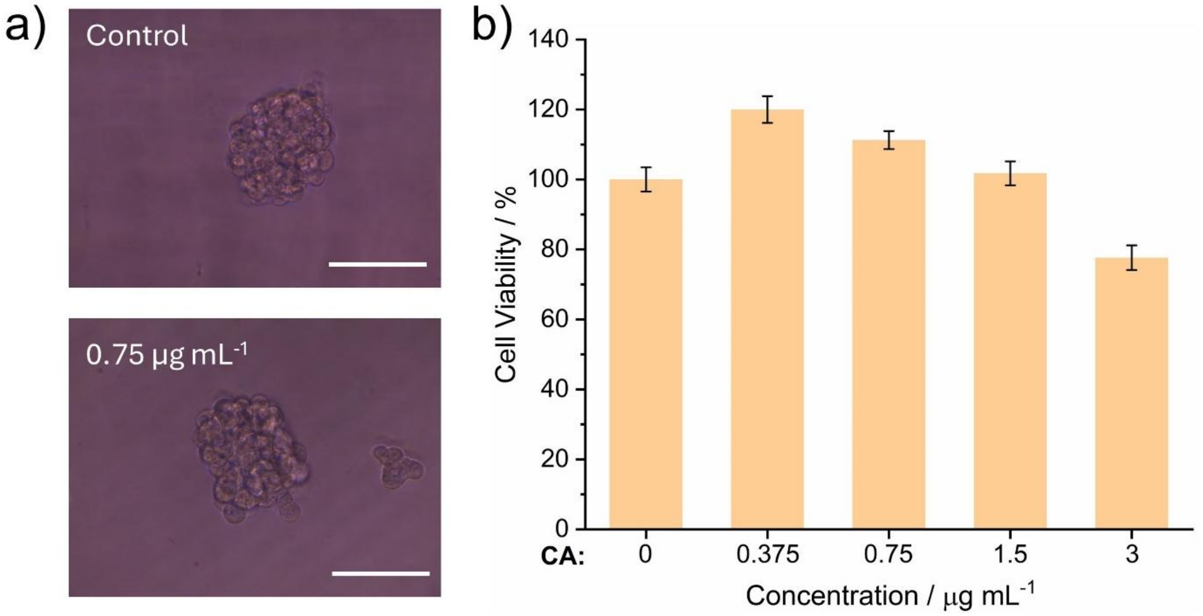

Figure S17. (a) Representative light microscopic imaging of HepG2 spheroids with and without 5 days incubation with 0.75 μg mL^{-1} CA. Scale bar: 100 μm. (b) Cell viability of HepG2 spheroids incubated with different concentrations of CA for 5 days. Data shown are mean of three biological replicates, error bars denote standard deviations.

Table S6. Table of IC_{50} of different samples in HepG2 spheroids after 5 days incubation (unreported values are the values that could not be determined in the used sample concentration range, n = 3).

Samples	IC_{50} (μg mL^{-1})
CA	/
CA@MOF-808(Fe)@PEG	14.99 ± 1.05
CA@MOF-808(Fe)@AuNP@PEG	13.61 ± 1.03
CA@MOF-808(Fe)@Mn@PEG	11.32 ± 1.03
CA@MOF-808(Fe)@AuNP@Mn@PEG	8.55 ± 1.05
MOF-808(Fe)@PEG	/
MOF-808(Fe)@AuNP@PEG	/
MOF-808(Fe)@Mn@PEG	41.43 ± 1.08
MOF-808(Fe)@AuNP@Mn@PEG	15.23 ± 1.05

S5. References

[S1] D. Zhang, E. N. Atochina-Vasserman, D. S. Maurya, N. Huang, Q. Xiao, N. Ona, M. Liu, H. Shahnawaz, H. Ni, K. Kim, M. M. Billingsley, D. J. Pochan, M. J. Mitchell, D. Weissman, V. Percec, *J. Am. Chem. Soc.* **2021**, *143*, 12315-12327.

[S2] Y. Ding, Z. Sun, Y. Gao, S. Zhang, C. Yang, Z. Qian, L. Jin, J. Zhang, C. Zeng, Z. Mao, *Adv. Mater.* **2021**, *33*, 2102188.

[S3] C.-H. Shen, Y.-H. Chen, Y.-C. Wang, T.-E. Chang, Y.-L. Chen, C.-W. Kung, *Phys. Chem. Chem. Phys* **2022**, *24*, 9855-9865.
